# Preclinical Studies and Drug Combination of Low-Cost Molecules for Chagas Disease

**DOI:** 10.3390/ph16010020

**Published:** 2022-12-23

**Authors:** Elena Aguilera, Carina Sánchez, María Eugenia Cruces, Belén Dávila, Lucía Minini, Florencia Mosquillo, Leticia Pérez-Díaz, Elva Serna, Susana Torres, Alicia Schini, Luis Sanabria, Ninfa I. Vera de Bilbao, Gloria Yaluff, Flavio R. Zolessi, Luis Fabian Ceilas, Hugo Cerecetto, Guzmán Alvarez

**Affiliations:** 1 Grupo de Química Orgánica Medicinal, Instituto de Química Biológica, Facultad de Ciencias, Universidad de la República, Montevideo 11400, Uruguay; 2 Laboratorio de Química Teórica y Computacional, Instituto de Química Biológica, Facultad de Ciencias, Universidad de la República, Montevideo 11400, Uruguay; 3 Laboratorio de Interacciones Moleculares, Instituto de Química Biológica, Facultad de Ciencias, Universidad de la República, Montevideo 11400, Uruguay; 4 Departamento de Medicina Tropical, Instituto de Investigaciones en Ciencias de la Salud, Universidad Nacional de Asunción, San Lorenzo 2169, Paraguay; 5 Sección Biología Celular, Facultad de Ciencias, Universidad de la República and Institut Pasteur de Montevideo, Montevideo 11400, Uruguay; 6 Ministerio de Salud Pública y Obra Social, Asunción 2511, Paraguay; 7 Laboratorio de Moléculas Bioactivas, Departamento de Ciencias Biológicas, CENUR Litoral Norte, Universidad de la República, Rute 3 km 363, Paysandú 60000, Uruguay

**Keywords:** Chagas disease, pre-clinically studied, drug combination, RMN metabolomics

## Abstract

Chagas disease is caused by the protozoan *Trypanosoma cruzi* (*T. cruzi*). It remains the major parasitic disease in Latin America and is spreading worldwide, affecting over 10 million people. Hundreds of new compounds with trypanosomicidal action have been identified from different sources such as synthetic or natural molecules, but they have been deficient in several stages of drug development (toxicology, scaling-up, and pharmacokinetics). Previously, we described a series of compounds with simple structures, low cost, and environmentally friendly production with potent trypanosomicidal activity in vitro and in vivo. These molecules are from three different families: thiazolidenehydrazines, diarylideneketones, and steroids. From this collection, we explored their capacity to inhibit the triosephosphate isomerase and cruzipain of *T. cruzi*. Then, the mechanism of action was explored using NMR metabolomics and computational molecular dynamics. Moreover, the mechanism of death was studied by flow cytometry. Consequently, five compounds, 314, 793, 1018, 1019, and 1260, were pre-clinically studied and their pharmacologic profiles indicated low unspecific toxicity. Interestingly, synergetic effects of diarylideneketones 793 plus 1018 and 793 plus 1019 were evidenced in vitro and in vivo. In vivo, the combination of compounds 793 plus 1018 induced a reduction of more than 90% of the peak of parasitemia in the acute murine model of Chagas disease.

## 1. Introduction

Chagas disease is recognized by the World Health Organization as a neglected tropical infectious disease in Latin America, where about eight million individuals suffer from this illness, and about 56,000 new cases per year are reported [1]. In addition, it is estimated that this disease causes the death of 10,600 individuals per year [2]. It is endemic in twenty-one Latin American countries and has become a global concern as a result of globalization and the mass migration of infected individuals [3]. As a result, Chagas disease is currently reported in 19 non-endemic areas including the European Union, Canada, the United States of America, Japan, and Australia [4]. This illness is caused by a monoflagellated parasite *T. cruzi*, and is transmitted mainly through the droppings of an animal vector, the Hemiptera insect (family *Reduviidae*, subfamily *Triatominae*) [5]. It can also be transmitted congenitally, through blood transfusions, heart transplantation, and orally through the consumption of food contaminated with the parasite [6]. Currently, commercially available drugs against Chagas disease are Nifurtimox (**Nfx**) and Benznidazole (**Bnz**), which are mainly effective in the acute or very early stages of the infection [7]. However, both drugs have many side effects, such as toxicity for the central nervous system and leukopenia, among others, and they are contraindicated in case of pregnancy due to their mutagenic effects [8,9]. Currently, efforts for handling this disease are focused on controlling the vectorial transmission but not on the drug development process to find more efficient drugs with low side effects [10]. In this last aspect, the drugs for this disease have to be inexpensive, since the population that suffers from this illness is mostly with low economic resources [11]. New molecules have been found as potential drugs for this disease, but their productions are complex and expensive [12,13,14,15]. An example of these molecules was Posaconazole (**Pos**) derivatives, which failed in advanced clinical stages, and which is ten times more expensive than the current commercial drugs [16,17].

The enzyme triosephosphate isomerase in *T. cruzi* (TcTIM) has become an interesting target for the design of new drugs because it is an essential enzyme used in energy production from glucose by the parasite [18]. TcTIM catalyzes the isomerization of glyceraldehyde-3-phosphate (G3P) to dihydroxyacetone phosphate (DHAP) in the fifth step of glycolysis. Notably, parasites are not viable in the absence of TIM. Thus, this enzyme is druggable, and it has been proposed as a possible target for the design of drugs for Chagas disease [19]. This is a constitutive enzyme in every existing living organism. TcTIM and *Homo sapiens* triosephosphate isomerase (HsTIM) share the same catalytic residues. However, 32 interfacial residues of TcTIM in parasites and HsTIM are different. Since both enzymes share 52% of homology, inhibitors that show specificity for the interface of the parasite’s enzyme could be selective for *T. cruzi* [20,21,22]. For this reason, many efforts have been pursued to design compounds capable of inhibiting selectively TcTIM without altering HsTIM [23,24]. Therefore, our group began searching for compounds that were able to disrupt the TcTIM interface, destabilizing the dimeric structure of the enzyme and making it inactive, interrupting the glycolytic pathway to stop the proliferation of the parasite [25]. In addition, the accumulation of DHAP due to TcTIM inhibition could lead to the formation of methylglyoxal, causing nonspecific glycation of macromolecules, leading to the disruption of metabolic pathways [26]. Finally, the excess of this substance ends up in the formation and accumulation of lactate [26]. 

A family of more than twenty symmetric diarylideneketones was designed as potential inhibitors of TcTIM [25]. Eight of them had an inhibitory concentration of 50% of the activity (IC_50_) less than 5 µM for TcTIM activity. In particular, compound **1019** (Table S1) showed an IC_50_ of 86 nM and was 12 times more active against epimastigotes of *T. cruzi* than the reference drug **Nfx**. Furthermore, two more compounds demonstrate moderate inhibition of this enzyme, compound **793** and **1018**, with an IC_50_ of 3.0 and 4.7 µM, respectively, but **1018** proved to be the most potent compound of our chemo-library against *T. cruzi* epimastigotes, with an IC_50_ of 40 nM (200 times more active than the reference drug **Nfx**). Interestingly, the selectivity against the human enzyme was probed with these compounds and no inhibition could be found at more than 10 times the effective dose [25].

Cruzipain (CP), the main cysteine protease present in all stages of the life cycle of *T. cruzi,* is an endopeptidase that, like other enzymes of this class, binds the substrate in an extended conformation, locating its side chains in binding subsites located in the active site of the protein [27]. The functions of CP are not yet fully defined but could include: lysosomal digestion of proteins, protection against the host’s immune response, a role in the penetration of trypomastigotes into the mammalian cell, and/or a role in the differentiation stages at different points in the parasite’s life cycle [28,29]. Studies carried out using cysteine protease inhibitors indicate the important participation of cruzipain in the survival of the parasite. These studies open the possibility of developing CP inhibitors as trypanosomicidal compounds [30,31,32]. 

Noteworthy, studies have revealed that polypharmacology for this disease can give successful results [33]. In 1988, the first study was carried out, where Ketoconazole (**Ktz**) was combined with another antifungal agent, Terbinafine (**Tbf**) [34]. According to the in vitro isobolograms results, **Ktz** and **Tbf** had a synergistic action in both epimastigotes and amastigotes [35]. The authors proposed that this potentiation is consistent with the fact that **Ktz** and **Tbf** act at different points in the ergosterol biosynthetic pathway and consequently amplify the effects [36]. Furthermore, the authors suggested that both drugs could be combined in the therapy of *T. cruzi* infections using fewer doses to avoid interference with the synthesis of steroids in the host. Some studies have been performed in clinical cases of Chagas disease (chronic asymptomatic patients), comparing the effect of the combination of **Bnz** with the antifungal **Pos** to the effect of each drug alone. The effect of **Bnz** alone was shown to be superior to **Pos**, although in 37% of cases its administration had to be suspended due to side effects, the combination did not show beneficial effects in therapy [37,38]. 

In the present study, we evaluate if a polypharmacological approach employing the compounds developed by our group, which have different mechanisms of action, could achieve better antiparasitic activity than when used in monotherapy.

## 2. Results and Discussion

### 2.1. Chemistry

The synthesis of these compounds was reported previously [25], but here we made little procedure modifications to obtain green chemistry conditions and better production yields. Then, we use 100% water instead of the mixture ethanol/water, and only 1 eq. of sodium hydroxide at 25 °C. Additionally, we scaled up from 1g to 100 g, with 85% of yield in the described condition. The purity until the end of the reaction using only filtration and a water washing, was more than 95%.

The highlights of the chemistry of these compounds are the low-cost production, the high product yield, and the simple and environmentally friendly production (see Supporting Material Section S1). Consequently, they can be scalable from grams to kilograms, the synthetic procedures are only one or two reaction steps. That is an important issue in the drug development process for a neglected disease because the cost of the treatment is one of the most important limitations.

### 2.2. Biological Evaluation

#### 2.2.1. Toxicology

Because under monotherapy our compounds demonstrated in vivo activity in the murine model of Chagas disease [39,40,41,42], we decided to complete a series of toxicity tests to assemble the toxicological profile at the preclinical stage. For this, we evaluated mutagenicity (for **1018**, **1019**), genotoxicity (for **793**, **1018**, and **1019**), teratogenicity (**793**, **1018,** and **1019**), and oral acute toxicity in mice (for **793**, **1018**, and **1019**) (Table 1). In the Ames test, compounds **1018** and **1019** were classified as non-mutagenic because they did not display significant differences in the number of revertant colonies of *S. typhimurium* compared with the negative control at five times the assayed doses (see in Supplementary Material, Table S2). In the genotoxicity assay, we also did not observe significant differences in the micronucleus appearance between mice treated with these compounds and the untreated animals used as a negative control. On the opposite, there was a significant difference between our compounds and cyclophosphamide (positive control for genotoxicity). While cyclophosphamide induced the generation of 36 ± 2 multimicronucleated cells (MnPE) per mouse, our compounds and the control generated only around 5 MnPE (Table 1).

**Figure 1:**
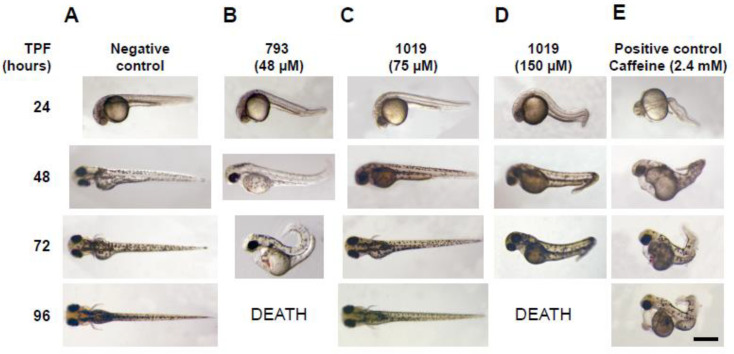
Fish Embryo Toxicity Test. Examples of zebrafish embryos treated with different compounds, or vehicle alone, from 6 to 96 h post-fertilization (hpf), are shown at key time points. (**A**) Embryos exposed to 1% *v*/*v* of dimethylsulfoxide (DMSO). (**B**) Embryos treated with compound **793** at 48 µM. (**C**) Compound **1019** at 75 µM. (**D**) Compound **1019** at 150 µM. (**E**) Embryos exposed to 2.4 mM caffeine (positive teratogenic agent). Scale bar: 0.5 mm. TPF: time post-fertilization.

**Figure 2:**
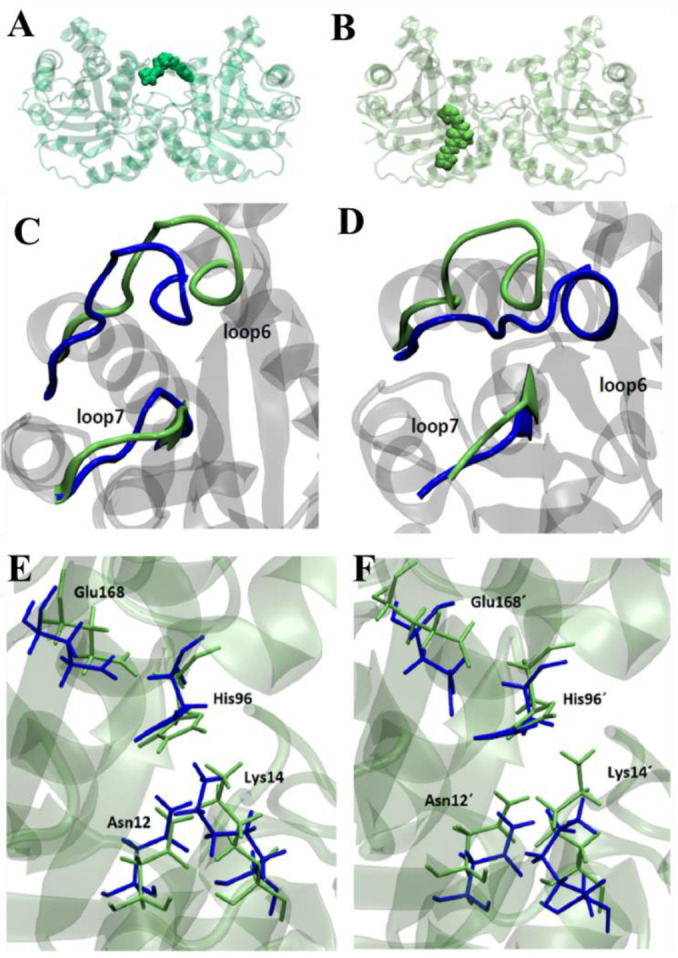
Site of the interaction of compound **1019** in TcTIM (**A**) after molecular docking and (**B**) after molecular dynamic studies, observing changes in the interactions from loop 6 to loop 7. Positioning of loops 6 and 7 in the presence of compound **1019** (green) and the absence of ligand (blue) (**C**) in monomer A and (**D**) in monomer B. Positioning of active site residues in the presence (green) and absence of compound **1019** (blue) (**E**) in monomer A and (**F**) in monomer B.

For the study of the toxicity of compounds **793**, **1018,** and **1019** in embryos, the Fish Embryo Toxicity Test (FET) recommended by OECD guides (Test Guideline 236) [43,44] was performed (Figure 1). For compounds **793** (Figure 2B) and **1019** (Figure 2C), serial doses of 3, 6, 12, 24, 48, 75, and 150 µM were used. The calculated LD_50_ value for compound **1019** in the 96 h exposure period of an early developing embryo was (100 ± 12) μM, a dose 167 times higher than its IC_50_ in the parasite (IC_50_ = 0.6 μM). The non-cyclic analog **793** displayed in the same assay an LD_50_ of 22 μM, which was four times its IC_50_ in *T. cruzi.* For compound **1018,** we only evaluated a single dose of 25 μM in a 96 h exposure period and we did not see any toxic effects in the embryos, and the selectivity was calculated as 625 times (Table 1). In addition, the bioconcentration factor that our group has demonstrated to exist in this type of assay [39] must be considered where the medium is aqueous and fat-soluble molecules are concentrated in the embryo along the time, as it can cause an exacerbation of toxic effects.

After verifying, through the studies indicated above, the low in vitro and in vivo toxicity and the potent trypanosomicidal activity of these molecules and following the steps of preclinical drug development, the acute oral toxicity in vivo in mice was tested. For this study, the compounds were administered orally in the same microemulsion that was used in the pharmacological study on the acute model of Chagas disease [42]. The initial number of animals is defined by the software AOT425 Stat and also depends on if there are LD_50_ of compounds with similar structures. Variable numbers of animals could be used if there is some toxicity observed during the first administrations. For Compound **793**, a dose of 550 mg/kg of body weight (b.w.) was started orally in three mice and then increased to a maximum dose of 2000 mg/kg b.w. until one of the trial termination criteria was met. In this case, one of the animals died 10 days post-administration. The LD_50_ calculated by the statistical program from these experiments was 2000 mg/kg b.w., the maximal dose being used for oral administration. As a result, compound **793** has low acute toxicity in these conditions. For compound **1019**, a maximum dose of 2000 mg/kg b.w. was started orally in five mice until one of the ends of the trial criteria was met. The LD_50_ calculated by the statistical program is greater than 2000 mg/kg b.w., the maximal dose assayed, demonstrating that compound **1019** also has low acute toxicity under the test conditions. The same results were obtained with compound **1018**. Throughout the test, during the 14 days, the mice were evaluated by the Irwin test and their masses were recorded. We observed that compounds **793**, **1018,** and **1019** did not show signs of acute toxicity in this model. Altogether, these molecules demonstrate a safe profile to be a drug candidate in the treatment of Chagas disease.

#### 2.2.2. Analyzing the Potential Mechanisms of Actions

To study the mechanisms of action of these molecules, this section describes a series of tests that evaluated possible molecular targets.

#### Triosephosphate Isomerase Mechanism of Inhibition

Previously, we demonstrated the capability of compounds **793**, **1018,** and **1019** to be an inhibitor of the triosephosphate isomerase recombinant enzyme of *T. cruzi* (TcTIM) [25]. Here, we describe molecular dynamics and metabolomics studies to supplement the information about this possible target. We have already shown that these compounds displayed high selectivity against the recombinant enzyme of *T. cruzi*, as we demonstrated when these compounds were tested against other TIM from a diverse panel of organisms (*Homo sapiens*, common cattle tick *Rhipicephalus microplus* [45], *Fasciola hepatica* [46], *Leishmania mexicana*, *Schistosoma mansoni*, and *T. brucei*). We did not see any TIM-inhibitions at 25 µM of those compounds, not even against the closely structure-related TIM from *T. brucei*. This not only demonstrates the selectivity but also demonstrates experimentally that these compounds cannot be interference compounds nor promiscuous inhibitors. 

In this work, we performed molecular dynamic studies, to simulate the in-solution motion of the interface, and how it affects the ligand–protein interaction. Starting from the docking of compound **1019**, where the compound interacts at the interface of the TcTIM, the dynamics were carried out under the same conditions. However, as it can be seen in Figure 2B, compound **1019** did not remain in the interface after the molecular dynamics protocols, but moved from it, passing through loop 6. Furthermore, the presence of this compound encourages loop 6 to open away from loop 7. This would not explain the inhibition of this compound, because when there is inhibition of TcTIM, this loop tends to close due to the occupation of the active site (Figure 2C,D). In addition, this compound generated changes in the position of the catalytic residues (Figure 2E,F). From the study of the mean squared distance of the fluctuations (RMSF), there was only one important difference at the level of loop 6 of monomer B (see Supporting Information Section S3). This change was reflected in the positioning of said loop 6 in this monomer. This change was due not only to its opening but also to the shift observed for loop 7 in the absence of a compound. Thus, compound **1019** could be affecting the catalytic site by the stiffening of the motion of loop 6. 

#### NMR Metabolomics Studies

This technique for quantifying internal or excreted metabolites by ^1^H-NMR, implemented in our research group, is simple since, unlike standard biochemical techniques, all metabolites can be identified in a single experiment. The signals of the metabolites identified in the culture medium by epimastigote of *T. cruzi*, Y strain, are shown in Table 2. Deuterated water was used as a principal solvent. Compound **1019**, the most potent TIM inhibitor, was evaluated at 0.15 µM to explore the ability of this compound to affect the parasitic energetic metabolism. It appeared that this compound could generate an accumulation of lactate into the parasite, as well as an enhanced excretion of glycine and succinate. The accumulation of lactate is related to an accumulation of DHAP, as a consequence of the TIM inhibition (see Supporting Material Section S4, ^1^H-NMR experiments of internal metabolites). Then, this lactate is transformed into glycine to be excreted from the parasite, to avoid its toxicity. The accumulation of succinate could be the result of the use of succinate as a co-transported metabolite in the secretion of glycine because glycine needs to be combined with succinate for the elimination of nitrogen [47,48].

When comparing the metabolism of glucose *vs* fructose, fructose is metabolized using a different enzyme than in glycolysis, bypassing the TIM enzymatic action. Thus, if the cell is in the presence of fructose and the absence of glucose, the parasite can generate energy from fructose. Therefore, if we inhibit the TIM, the parasite should live without problems in the presence of fructose. To confirm this theoretical observation, we grew the parasite in the absence of glucose and with a high concentration of fructose, and we recalculate the IC_50_ of **1019** against *T. cruzi*. The IC_50_ under fructose metabolism appeared 4 times higher than under glucose, confirming that one of the principal targets of this compound is TcTIM. At the opposite, the IC_50_ of compound **1018**, a moderate TcTIM inhibitor, did not change significantly under the fructose metabolism, suggesting another principal target. This observation correlates with the significant difference between its IC_50_ in epimastigotes and in TcTIM inhibition, with the IC_50_ in epimastigotes being 125 times higher than the IC_50_ on the TIM inhibition. 

#### 2.2.3. Mechanism of Death

It has been described that it is possible to know if a cell dies by necrosis or apoptosis through changes in lipid turnover and membrane structure by ^1^H-NMR [49]. In our group and in previous works, it has been described that, in cell systems whose death is by apoptosis, the ratio between the intensities of methylene (CH_2_) (1.3 ppm) and the methyl group (CH_3_) (0.9 ppm), due to membrane lipids, increases [50]. However, for necrosis, it is observed that the choline signal (3.10–3.30 ppm) disappears. The cellular origin of these resonances is mainly due to the majority presence of triacylglyceride side chains. In the treatment with compound **793**, it was observed that the parasites were probably undergoing apoptosis and late necrosis as the choline signal, present in the control, disappeared (Table 3) and the CH_2_/CH_3_ ratio increased. In the case of compound **1019** and **Nfx**, it showed a disappearance of the choline signal with no increase in the CH2/CH3 ratio, suggesting that necrosis was the principal cause of death. For compounds **1018** and **Bnz**, neither apoptosis nor necrosis characteristic changes in the ^1^H-NMR spectra were observed.

Additionally, we evaluated the viability of treated parasites by flow cytometry analysis after incubation with methylated acetoxycalcein (CA-AM) and propidium iodide (IP). Parasites were incubated for 6 h and 24 h with **Nfx**, **Bnz**, **793**, **1019,** and **1018** at a concentration corresponding to 20 × IC_50_ (100 µM, 12 µM, and 0.8 µM for compounds **793**, **1019,** and **1018**, respectively). Since the methodology used to determine the IC_50_ is based on 5 days of incubation, doses 20 times higher were used to analyze cell viability in shorter times. Similarly, these compounds’ concentrations were used to determine the death mechanism involved at the aforementioned times. 

**Figure 3:**
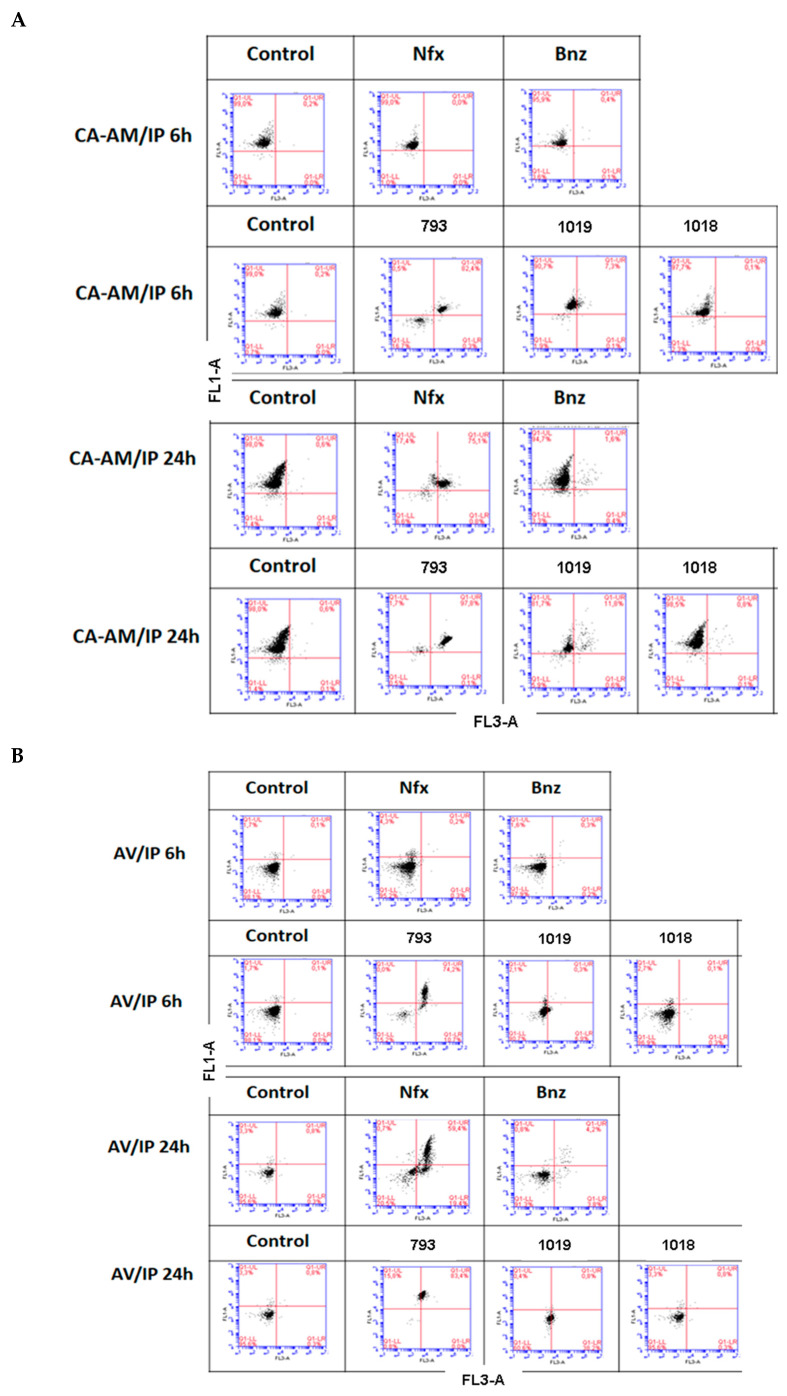
Analysis of the viability and mechanism of death by flow cytometry in parasites at 6 and 24 h of incubation at a 20 × IC_50_ concentration of **Nfx**, **Bnz**, **793**, **1019**, **1018**. (**A**) CA-AM/IP labeling. (**B**) AV/IP labeling.

Based on the results of the CA-AM/IP labeling, 82% and 98% of double-labeled parasites were observed for compound **793** after 6 h and 24 h of incubation, respectively, which is compatible with cell death due to a late apoptosis/necrosis mechanism [51]. The CA-AM^+^/IP^+^ positive phenotype may be explained by an increase in the permeability of the plasma and nuclear membranes without affecting their integrity. For compound **1019**, a possible late apoptotic cell death was also observed, since a slight shift of cells towards the CA-AM^+^/IP^+^ zone was evidenced in the presence of the compound at 6 h (7.3%) and 24 h (11.8%) (Figure 3A). For the reference drug **Nfx**, at 24 h, double labeling of the CA-AM^+^/IP^+^ parasites was observed, indicating that a late apoptosis mechanism is involved. No changes were observed for parasites treated with **Bnz** or **1018** (most cells are CA-AM^+^) 

**Figure 4:**
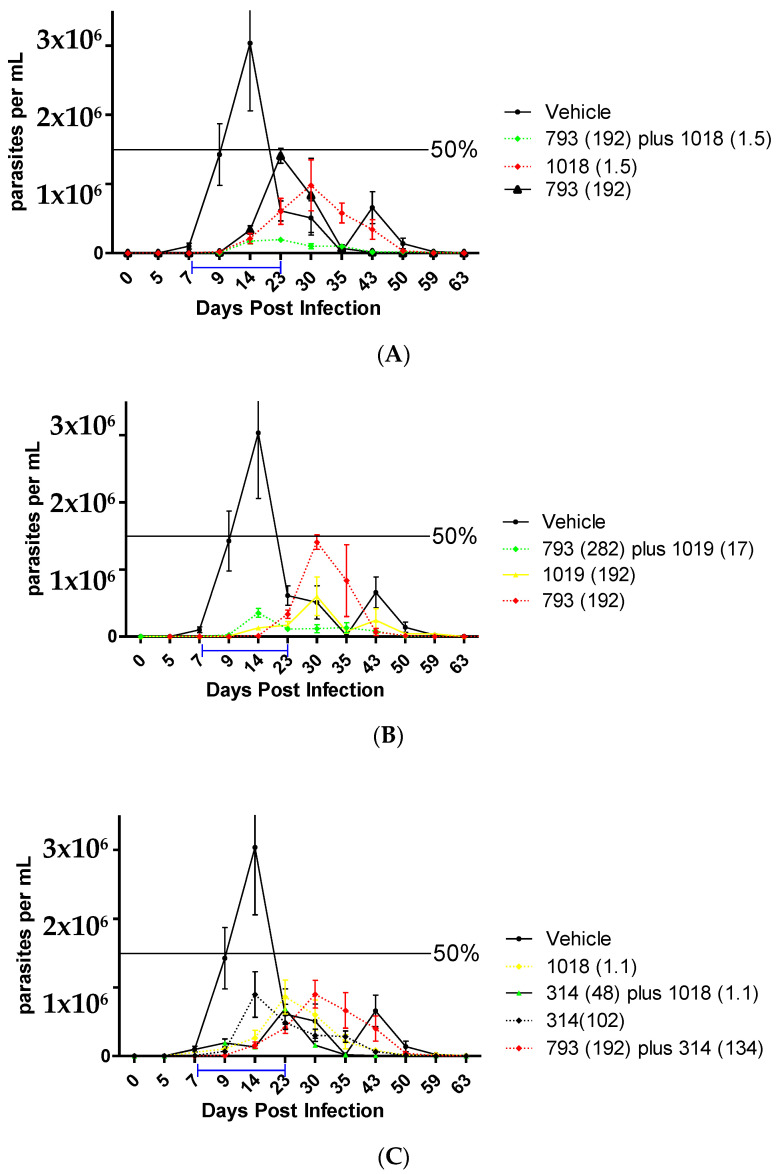
In vivo studies analyzing polypharmacology, using a murine model of Chagas disease. (**A**) Combination of **793** at 192 µmol/kg b.w./day with **1018** at 1.5 µmol/kg b.w./day for 14 days. (**B**) Combination of **793** at 282 µmol/kg b.w./day with **1019** at 17 µmol/kg b.w./day. (**C**) Combination of **314** at 134 µmol/kg b.w./day with **793** at 192 µmol/kg b.w./day and **314** at 48 µmol/kg b.w./day with **1018** at 1.1 µmol/kg b.w./day. The blue bar indicates the treatment period. The colors of the plot indicate the grade of efficacy (green as good, yellow as medium, and red as low). The doses used are indicated in µmol/kg b.w./day in parentheses for each compound.

To deepen the cell death mechanism involved after treatment with the aforementioned compounds, Annexin V (AV) and IP labeling was also assayed and followed by flow cytometry (Figure 3B). For parasites treated with compound **793**, double AV^+^/IP^+^ labeling was observed, which is in agreement with the results shown in Figure 4A and with the ^1^H-NMR experiments, which would indicate that the death mechanism induced by this compound would be late apoptosis (74% at 6 h vs. 84% at 24 h). For compound **1019**, necrotic cell death may be suggested since an increase from 7% to 38% IP^+^ parasites was observed after 6 h and 24 h, respectively, with no AV labeling. For compound **1018** treatment, no significant differences were observed compared to untreated parasites, so it cannot be concluded. For treatment with the reference drug **Nfx**, a late apoptotic/necrotic mechanism is involved in cell death due to the double AV^+^/IP^+^ labeling (59%) at 24 h. Finally, no apoptosis, late apoptosis, or necrosis was observed for the other reference drug, **Bnz**.

#### 2.2.4. In Vitro Metabolic Stability

The qualitative analysis of the in vitro metabolization by the rat hepatocyte microsomal (MF) and cytosolic (CF) fractions of compounds **793**, **1019,** and **1018** was performed using chromatographic techniques (reverse phase high performance liquid chromatography and thin-layer chromatography). For this, the metabolization was monitored at different times (0, 30, 60 min and 4 h). In both MF and CF of hepatocytes treated with compounds **793** and **1019**, it was observed that after one hour, more than 50% (t_1/2_ < 60 min) of the compounds were metabolized to more hydrophilic compounds. For these compounds, it was observed the appearance of new products of metabolization over time. Finally, compound **1018** was the less modified one, since after 4 h (a time suggested in the drug development guidelines for optimal metabolic stability) no new compound was observed. Regarding the structure and metabolism, the heterocycles (thienyl vs. furyl) could be responsible for these different stabilities: the furyl ring would metabolize to an open cycle version, making this system more labile in front of the hepatic metabolization [52]. 

#### 2.2.5. Trypanosomicidal Activity in Other Forms of the Parasite

Additionally, these compounds were tested against the trypomastigote form of *T. cruzi* (strain Y). In this system, the IC_50_ for compounds **793** and **1019** was measured at 25 ± 5 µM. Thus, despite these forms having a high dependence on energy production, with glycolysis being one of the principal pathways, the TIM inhibitors seem to be less active against trypomastigotes. The reasons for this observation can be the slow action of this mechanism of inhibition, or because these forms of the parasite can excrete metabolites more easily than the other forms. That can lead to a decrease in the accumulation of the toxic metabolic intermediates, leading to a concomitant decrease in the trypanosomicidal effect of our compounds. Interestingly, compound **1018**, which was the most potent in the epimastigote form with an IC_50_ of 40 nM, remains the most efficient in the trypomastigote form with an IC_50_ of 7 ± 2 µM. Although not in the same range, this compound seems to remain the most potent against any form of the parasite. 

#### 2.2.6. Compound Combinations and In Vitro Isobolograms

The combination of different molecules that act in different pathways of the metabolism of the parasite was studied, seeking the complete cure of the disease, reducing doses, shortening treatment times, and/or reducing the doses of the current treatment. For this, compounds **793**, **1019**, **1018**, **314**, **1260**, and **Bnz** were combined. It is known that **Bnz** acts by modifying macromolecules through covalent bonds with the parasite components; or the association with DNA, lipids, or proteins [53]. Compounds **793** and **1019** are known to be inhibitors of TcTIM. Compound **1018** moderately inhibits TIM but is the molecule that best inhibits the growth of the epimastigote and trypomastigote forms of *T. cruzi*. Compound **314** is known to be an inhibitor of cruzipain. Compound **1260** is not an inhibitor of cruzipain but could be an inhibitor of glucose 6-phosphate dehydrogenase [54].

The corresponding in vitro isobolograms were carried out and demonstrated synergism for the combinations: (i) **793** + **Bnz**; (ii) **793** + **1018**; (iii) **793** + **1019**; (iv) **793** + **314**; (v) **1018** + **314** (Table 4). Additionally, the combination between **Bnz** and **314** was additive and all the combinations with **1260** were antagonists (Table 4).

#### 2.2.7. In Vivo Studies in the Acute Murine Model of Chagas

Given the promising results obtained by compound combinations in vitro and the low unspecific toxicity of these compounds, it was decided to continue with the in vivo pre-clinical proof-of-concept in the acute murine model of Chagas disease of these combinations. Previously, we demonstrated the grade of the efficacy of these compounds in monotherapy [25,40].

Compound **1018** was the most active in vitro, in the epimastigote form of *T. cruzi*, and also showed a low acute oral toxicity in vivo. However, the low survival of the mice in the monotherapy treatment at 192 µmol/kg b.w./day in the acute murine model of Chagas disease indicated either a lack of efficacy in infection condition or that the multiple doses could lead to toxic effects. Thus, we decided to work with compound **1018** always in combination with other compounds and at lower doses than the one previously tested (192 µmol/kg/day) [25]. For this, we selected the combination of **1018** with **793** according to the optimal in vitro molar ratio between these two compounds (Table 4), which is 125 moles of compound **793** for every 1 mole of compound **1018** and thus is compatible with the use of a lower dose for **1018**. Therefore, a microemulsion of **793** plus **1018** was prepared at a dose of 192 µmol/kg b.w./day of compound **793** plus a dose of 1.5 µmol/kg b.w./day of **1018**. Similarly, the combination of **1018** and **314** allowed the use of **1018** in a very low proportion (Table 4), i.e., 133 moles of compound **314** for every 1 mole of compound **1018**. It was also decided to work with the compounds **793** plus **314** because they have a value of FICI = 0.75 and both compounds have different bio-target. Taking into account the optimal molar ratio from its IC_50_, each mole of compound **793** needs approximately 1.2 moles of **314** (Table 4). Therefore, a combination of compounds containing 192 µmol/kg b.w./day of compound **793** plus 132 µmol/ kg b.w/day of **314** (ratio of 1.4/1) was used. Finally, the last analyzed combination, which showed a synergetic behavior, was **793** plus **1019,** using doses of 282 µmol/ kg b.w/day for the first compound plus 17 µmol/ kg b.w/day for the second one.

**Figure 5:**
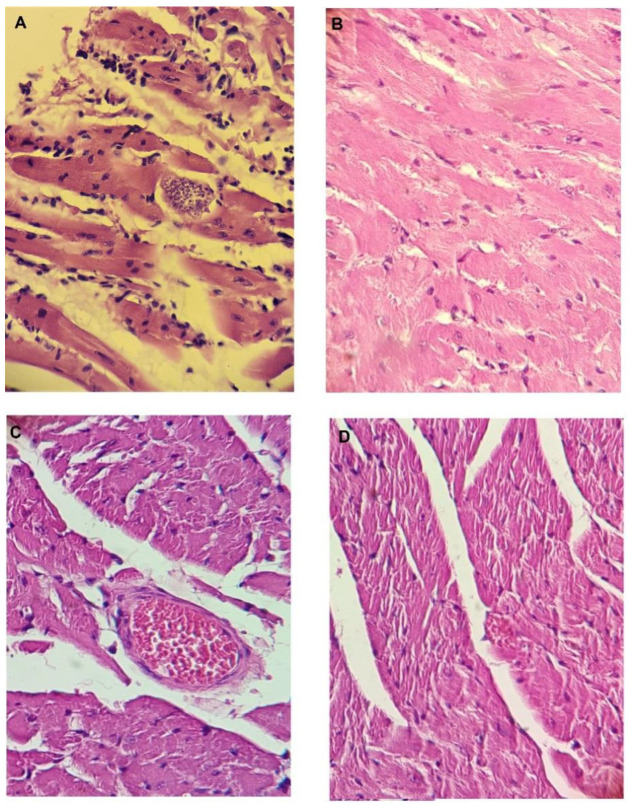
Heart tissue sections of animals infected with *Trypanosoma cruzi*: (**A**) untreated animals, the black arrow indicates the amastigote’s nests. (**B**) Animals treated with combination **793** + **1018** (according to doses shown in (**A**)). (**C**) Animals treated with combination **793** + **1019** (according to doses shown in (**B**)). (**D**) Animals treated with **Bnz**. Hematoxylin–eosin staining, 40× magnification.

The best in vivo activity was the combination of **793** (192 µmol/kg b.w./day) plus **1018** (1.5 µmol/kg b.w./day) (Figure 5A) with a 90% of reduction of the parasitemia peak compared to vehicle-treated animals, and 100% of the animal survive (Table 5). In this combination, we reduced the doses of **1018** to a concentration thirty-two times lower than the effective dose of **Bnz** (48 µmol/kg b.w./day). Additionally, the mechanism of death by flow cytometry changes from necrosis to apoptosis under this combination (see Supporting Material Section S5). In addition, the fact that a combination 1 to 1 of these compounds was not better than the monotherapy, this copes with the effect observed at the highest doses in monotherapy of **1018,** for which some non-specific toxicity appears. The other effective combination was **793** (282 µmol/kg b.w./day) with **1019** (17 µmol/kg b.w./day, corresponding to one-third of the effective dose of **Bnz)**, with a 65% of reduction of the parasitemia peak (Figure 4B).

The other combinations, **314** + **793** and **314** + **1018**, did not show significant improvement in the in vivo trypanosomicidal effect compared with the monotherapy (Figure 5C and Table 5). Thus, we observed a good correlation between the optimal dose ratios calculated in vitro and the in vivo behaviors. When an in vivo combination was used in a different ratio than the optimal determined in vitro, we did not observe an improvement of the efficacy and vice versa.

In addition, the ex vivo histopathological analysis of selected organs from the animals treated with the combination of **793** plus **1018** and **793** plus **1019** was performed at the end of the experiment. The animals were sacrificed at day 60 and the spleen, kidneys, liver, intestine, and heart of mice from each group were extracted to study the effect of the treatment in the typical organs related to *T. cruzi* infection. Histopathological changes in these organs of untreated and treated animals were observed with a light microscope after staining tissues with hematoxylin and eosin. The best results were observed in the heart as shown in Figure 5. The heart tissue from infected untreated animals showed pronounced mononuclear inflammatory infiltrates in the pericardium and between myocardial fibers, in addition to a significant number of amastigotes and amastigotes nests (Figure 5A). On the contrary, a relevant and significant decrease in these parameters was observed in the tissues of the treated infected animals (Figure 5B–D).

## 3. Material and Methods

### 3.1. Chemistry

The herein-studied compounds were selected from our chemical collection. The entire compounds reach more than 95% of purity. Synthesis and characterization of the compounds are available in the Supplementary Information.

### 3.2. Mutagenicity Tests (Ames Test) 

The mutagenic capacity of the compounds was determined from a 2 × 10^9^ CFU/mL culture in Oxoid No. 2 medium in the exponential phase of *Salmonella typhimurium* strains TA98, T100, T102, TA1535, and TA1537 (hisD3052, histidine-dependent) [42]. Five serial dilutions to the third in DMSO were evaluated, starting from the maximum non-toxic dose estimated from the results of the toxicity test carried out initially. Positive controls were performed with 4-nitro-*o*-phenylenediamine (20 µg/plate) in the case of mutagenicity without activation. DMSO (50 µL/plate) and phenotype control (resistance to ampicillin, tetracycline, crystal violet, and sensitive UV light) were used as negative control. Compounds were incubated for 1 h at 37 °C in 2 mL of an agar solution containing 200 µL of histidine solution [(0.5 mM)/biotin (0.5 mM)]. It was then grown on glucose minimal agar for 48 h at 37 °C. When the effect of the metabolization on the compounds was studied, the S9 fraction (Gibco™, Thermo Fisher Scientific, Grand Island, NY, US) counted and expressed as the mean of duplicates ± standard deviation. The product was considered mutagenic when the number of revertant colonies doubled that of the negative control in at least two consecutive doses. The preparation of the bacterial suspension, the dissolution of the S9 fraction, the product, and the first part of the test (until the tubes with all the solutions were transferred to the stove for incubation) was carried out under sterile conditions, in a laboratory laminar flow and with sterile material (autoclave at 121 °C for 20 min or maintaining the manufacturer’s sterility). The addition of the histidine/biotin solution and the seeding of the plates were carried out under an upward air current generated by a burner.

### 3.3. Teratogenicity in Zebrafish Embryos

Fish maintenance and embryo production: zebrafish lines were kept under controlled conditions, in an automated ZebTec (Tecniplast, Milan, Italy) stand-alone system at 28 °C, 500 µS/cm^2^ conductivity, pH 7.5, and fed with dry and live food (*Artemia salina*) three times a day, following accepted protocols and under the approval of the local and national ethical committees, Approval number 009-19 5/6/2019 (Comisión Honoraria de Experimentación Animal, Universidad de la República, CHEA-UdelaR, and Comisión Nacional de Experimentación Animal, CNEA, Uruguay). The SAT (Sanger AB Tübingen) wild-type line used for most experiments was obtained from the Zebrafish International Resource Center (http://zebrafish.org, access on 1 March 2018) (Eugene, OR, USA). Embryos obtained from natural crossings were cultured at 28.5 °C in system water and methylene blue (1 ppm) as a fungistatic. 1-Phenyl-2-thiourea (0.003%) was added to system water to inhibit melanogenesis in embryos destined for microscopic imaging. The eggs were collected, and the unfertilized ones were discarded. Fertilized eggs were incubated at 32 °C for about 5 h, where they are in a developmental stage of 75 % epiboly. At this stage, the membrane that protects the embryo (chorion) was manually removed. This was completed on a 1 % agarose plate and medium for embryos. Two 96-well plates with a curved bottom were prepared. Each well contains 200 μL and was arranged as follows: 16 wells with the negative control corresponding to 1% DMSO in aquarium water, 16 wells with the positive control corresponding to 2.4 mM of caffeine in reverse osmosis water, and 16 wells for each of the 5 different concentrations of the tested compound dissolved in 1% DMSO. Dechorionated embryos were transferred to the plate with a Pasteur pipette after 6 h post-fertilization (hpf). One embryo was placed per plate, and it was verified that it had not suffered any injury when it was transferred. After preparing the plates, the embryos were evaluated. For this, they were placed in a humid chamber to avoid the evaporation of the compounds and were incubated at 28 °C. At 24 h, the first observation was made and half of the medium in each well was replaced to ensure that the concentrations remained constant during the 96 h of exposure. To do this, 100 µL of medium was removed from each well and replaced with 100 µL of fresh medium. The embryos were observed under a stereoscopic microscope, one by one with special attention to the characteristics that allow discriminating positives from negatives (see below), the positive ones being considered as a sign that the compound shows certain toxicity for the embryo. In addition, any other type of abnormality that may indicate teratogenicity was recorded. The same was performed every 24 h until 96 h of exposure where the test was considered finished. The embryos were sacrificed in 70% ethanol. Those embryos that displayed at least some of the following characteristics were classified as positive (affected) embryos: embryo coagulation, lack of heartbeat, lack of somite formation, and no detachment of the tail. The curve obtained was fitted to a sigmoid using the OriginLab 8.5 program. Then the value of 50% positive embryos was extrapolated and the corresponding concentration value was defined as the LD_50_. Some other types of observed anomalies were also registered. For the validation of the test, there must be a survival rate greater than 90 % in the negative control, and a mortality rate greater than 40 % at 96 h of exposure in the positive control (caffeine at 2.4 mM) [39].

### 3.4. Acute Oral Toxicity In Vivo in Mice (Up and Down Test)

CD1 and Balb/c mice, 3 months old with an approximate weight between 18–20 g, were used, using 2 mice for each group following accepted protocols and under the approval of the local and national ethical committees No. 2014/4/1LQO (Comisión Honoraria de Experimentación Animal, Universidad de la República, CHEA-UdelaR, and Comisión Nacional de Experimentación Animal, CNEA, Uruguay). The compounds were administered orally using an intragastric cannula with the indicated vehicle (for all in vivo studies, the compounds were used in the vehicle previously described [42]) at the doses recommended by the Food Drug Administration. The main test consists of a single ordered dose progression in which animals are dosed, one at a time, at a minimum of 48 h intervals. The first animal receives a dose a step below the level of the best estimate of the LD50. If the animal survives, the dose for the next animal is increased by (a factor of) 3.2 times the original dose; if it dies, the dose for the next animal is decreased by a similar dose progression. (Note: 3.2 is the default factor corresponding to a dose progression of one-half log unit). After administration, the animals were observed for 24 h and if there were no signs of toxicity, the administered dose was doubled. They were observed for a further 24 h, then if there are no signs of toxicity, a ten times higher dose was administered (up to a maximum of 2000 mg/kg). Mice were observed for 24 h after that last administration and were left untreated with food and water for a week. As a measure of toxicity, the general appearance and behavior of the animal and the daily weight change are observed. The experiment ends at 14 days post-administration, if there are no signs of toxicity, the (LD_50_) is the administered dose, if not, the software AOT AOT425 Stat program recommendation was followed. At the end of the experiment, the mice were sacrificed by cervical dislocation, a necropsy was performed, and the organs were macroscopically observed to visualize signs of toxicity. For the prediction of the LD_50_ of the compounds, online software was used (http://tox.charite.de/protox_II/, access on 1 October 2020 ) [55].

### 3.5. Genotoxicity Evaluation by In Vivo Micronucleus Test in Mice 

The protocol was approved by the Paraguayan ethics committee for animal experimentation (No. IORG0010088). The acute oral treatment was carried out in 3-month-old CD-1 mice with 150 mg/kg of the compound to be studied. Three treatment groups were used; GROUP I: negative control, treated with 200 μL of vehicle, GROUP II: treated with 150 mg/kg of the compound to be evaluated, GROUP III: positive control, treated with 50 mg/kg of cyclophosphamide. The treatment of each group was repeated five times, totaling 5 animals per concentration. The route used in the administration of the negative control and the compound (groups I and II) was oral, using an esophageal cannula and in group III the route was intraperitoneal. The intervention was carried out in two doses: 24 and 48 h before sacrifice, except for cyclophosphamide, which was administered in a single dose, 24 h before sacrifice. At the end of the experiment, the femurs were harvested, and the red bone marrow was removed with fetal bovine serum, kept at 37 °C. The material was homogenized and transferred to a conical centrifuge tube. Then, the tubes were centrifuged at 1000 rpm for 5 min, the supernatant was discarded, and the samples were prepared with the remaining cells by fixing them in absolute methanol for 5 min. Samples were stained with 4 % Giemsa stain for 3 min and analyzed under an immersion light microscope. For each treated animal, 1000 polychromatic erythrocytes (EPCs) were counted, including those that presented micronucleus (EPCMNs). The ratio of polychromatic erythrocytes (EPC) vs. normochromatic erythrocytes (ENC), upon counting 100 cells, was also evaluated. The proportion of EPCMNs was calculated for each group, and the treated groups with the compound and cyclophosphamide were compared with the negative control group. The statistical analysis was carried out from the individual values of the evaluated parameters, calculating the mean values and their standard deviations for each of the experimental groups. The data were processed using the statistical analysis software SPSS 21.0. ANOVA (*p* < 0.05) [41].

### 3.6. Molecular Dynamics Studies in TcTIM

To predict the binding site of compounds in TcTIM (PDB ID 1TCD), flexible ligand docking was performed using a 124 × 126 × 126 dot grid box with a grid space of 0.603 Å to cover the entire protein surface (Blind Docking). The grid box was centered on the macromolecule. Once the binding site was determined, the binding free energies were refined using a smaller 60 × 60 × 66 dot grid box with a 0.375 Å spacing, now centered on the ligand-binding site. Results that differ by less than 2.0 Å in square root deviation were pooled in the same group. The conformation with the lowest binding energy was chosen from the most populated group and the corresponding ligand–protein complex was used for further molecular dynamics studies. All docking calculations were performed with the AutoDock 4.2 software package using the Lamarckian genetic algorithm. A population size of 150 individuals and 2.5 × 10^6^ energy assessments were used for 50 independent search runs. Default values were used for the rest of the parameters. This procedure was followed by 100 ns of ligand–protein molecular dynamics as described previously using the General Amber Force Field (GAFF) for ligands and AMBER *ff03.r1* force field for TcTIM [56,57]. The molecular dynamics was performed with the *pmed.cuda* module in AMBER 14 [58]. The partial charges of the compounds were derived with the RESP fitting procedure [59] by HF/6-31G* single-point calculation on the optimized ligand structure. Trajectories analyses were carried out using the *cpptraj* module in AmberTools15 and the VMD program was used for visualization [60]. The stability of TIM without ligand and the corresponding inhibitor–enzyme complexes were verified from the mean square deviation (RMSD) and the flexibility of the systems was evaluated from the mean square fluctuations (RMSF). Energy-minimized structures were used as the reference structure for RMSD calculations. Solvent-accessible surface area (SASA) was calculated on selected interfacial residues for apo-TcTIM and TcTIM.

### 3.7. Metabolomics Studies of T. cruzi Using ^1^H-NMR

To evaluate the changes in the metabolic profile induced by the compounds we studied, 1 mL of 10 million epimastigotes, Y strain, was incubated in a culture medium (BHI-tryptose), at 28 °C for 48 h with the compound of interest. At zero hour of the experiment, before preparing the 10 million parasites/mL, the system was centrifuged at 3000× *g*, 10 min, the medium was discarded, and it was replaced by fresh medium. A “Culture medium control” was included in each test, containing 1 mL of culture and 0.5% of DMSO; corresponding to the solvent in which the compounds were evaluated, in the same concentration that was added by the volume of compound. The compounds were used at 10 times their IC_50_. After 48 h, the culture was centrifuged at 3000× *g*, 10 min, and 500 µL of the supernatant was taken and added to a 5 mm NMR tube. Then 10 µL of dimethylformamide (DMF) (internal standard) and 90 µL of deuterated water (D_2_O) were added. The spectra were acquired by making a total of 64 scans and were processed: “Full FT” was applied to the FID and the spectrum was referenced concerning EtOH at 1.17 ppm. The results were expressed as a percentage relative to the DMF in the “Culture medium control”. A positive percentage means an increase in the excretion of the metabolite compared to the control without treatment, and a negative percentage implies a decrease in the excretion of the metabolite compared to the control. For the study of internal metabolites, the parasites under the same conditions described above were washed three times with PBS and resuspended in 0.6 mL of deuterated water and chloroform, the mixture was sonicated for 5 min at maximum power and then centrifuged at 10,000× *g* for 5 min. The supernatant, aqueous upper phase, was taken into an NMR tube and analyzed on 500 MHz equipment, with the function of suppressing water and proteins.

### 3.8. Study of the Mechanism of Death by ^1^H-NMR in T. cruzi

Preparation of the cell sample: 10 million parasites/mL, Y strain of *T. cruzi* in the epimastigote form, were inoculated into 24-wells plates at 0.6 mL/well. Each plate was incubated with the compounds to be evaluated at the desired concentration (10 times their IC_50_); in addition, a control plate was included where the solvent in which the compounds were dissolved was inoculated in each well (24 in total). After incubation with the compounds, the parasites were collected from each well in a 15 mL conical tube, centrifuged at 3000 × g, for 10 min, and the supernatant was discarded. The pellet was washed 3 times with sterile PBS and resuspended in 500 µL thereof. Finally, 500 µL of PBS was added to the pellet in a 5 mm NMR tube and 90 µL of D_2_O was added to it. The parasite suspension was homogenized before introducing the tube into the NMR equipment. The spectra were acquired by making a total of 128 scans and were processed as follows: on the FID, “Full FT” was applied. The manual phase was adjusted (applying for “zero order”), the baseline was corrected by applying “Full Auto” and the spectrum was referenced. The EtOH signal (1.170 ppm) was used as an internal standard.

### 3.9. Study of the Mechanism of Death by Flow Cytometry

For cell death mechanism studies induced by the compounds, the Alexa Fluor^®^ 488 annexin V/Dead Cell kit (Thermo Fischer Scientific, Waltham, MA, USA) was used. One million parasites/mL of the epimastigote form of *T. cruzi*, Tulahuen 2 strain (DTU Tc VI) (in exponential phase under aerobic conditions) were incubated with the compounds (evaluated at 20 × IC_50_ for 6 and 24 h), then washed 3 times with 1X PBS at 3000× *g* for 10 min and finally resuspended in 100 µL of annexin binding buffer (50 mM HEPES, 700 mM NaCl, 12.5 mM CaCl_2_, pH 7.4). Parasites were incubated for 15 min with 2 µL Annexin V (AV) 5 mg/mL (Alexa Fluor^®^488) and 1 µL propidium iodide (IP) 1 mg/mL. Parasites were immediately analyzed on an Accuri C6 flow cytometer (BD Bioscience, Franklin Lakes, New Jersey, U.S.). Two parameters analysis was performed using a 533/30 nm signal detector (FL1) for AV and a 670 nm long pass emission signal detector (FL3) for IP. Ten thousand events were analyzed for each of the two independent experiments. Data were analyzed using BD CSampler software (BD Bioscience). Untreated parasites were used as a control. For the positive control of apoptosis and necrosis, the parasites were treated with hydrogen peroxide (H_2_O_2_) 50 µM and 100 µM for 2.5 h, respectively. These single-spot controls were used to calculate color compensation for flow cytometry.

Viability studies of parasites by flow cytometry: The feasibility studies were carried out by evaluating the esterase activity of the parasites using Acetoxymethylated Calcein (CA-AM) and IP (Thermo Fisher Scientific). Untreated parasites (used as control) and compound-treated parasites (1 × 10^6^ parasites/mL), evaluated at 20 × IC_50_ for 6 and 24 h, were harvested by centrifugation after 6 and 24 h of incubation, washed three times and were resuspended in 0.1 mL of 1X PBS containing 0.1 mM of CA-AM and 1 mg/mL of IP. The samples were incubated for 45 min at room temperature for CA-AM and 15 min for IP. Subsequently, they were immediately analyzed by flow cytometry with a 533/30 nm filter (FL1) for CA-AM and a long-pass filter of 670 nm (FL3) for IP. Fluorescence intensity for 10,000 events was acquired from two independent experiments and data were analyzed using BD CSampler software (BD Bioscience).

### 3.10. Stability Studies with Microsomal and Cytosolic Fractions of Rat Hepatocytes

For the determination of the stability in the different fractions, cytosolic and microsomal, rat hepatocytes protein fractions were purchased by Sigma^®^ and used according to the previously reported protocol [61]. The protein concentration in the different fractions was determined by the bicinchoninic acid assay (BCA), as suggested in the manufacturer’s manual (Sigma^®^, St. Louis, MO, US). The final concentration of the molecules in the aqueous medium was 400 µM and prepared from stock in DMSO of 40 mM. The solutions were homogenized and incubated at 37 °C for 10 min, 30 min, 1 h, 2 h, 3 h, and 4 h (by TLC). Incubation at 37 °C was performed in a reaction volume of 1 mL containing 2.5 µL of 30 mM magnesium chloride (MgCl_2_); 2.5 µL of 40 mM nicotinamide adenosine dinucleotide phosphate (NADP^+^); 5 µL of 350 mM glucose 6-phosphate (Glu6P); 5 µL of glucose 6-phosphate dehydrogenase (Glu6PD) 50 U/mL, 5 µL of the stock of 40 mM compounds and the volume of the phosphate buffer (pH = 7) was defined so that cytosolic (CF) and microsomal (MF) fraction present a final protein concentration of 0.1 mg/mL. After that, the stability of compounds **793**, **1018,** and **1019** in the CF and MF of rat hepatocytes was evaluated by HPLC at different times: 0, 30, and 60 min. At the end of the incubation, the compound was extracted with 200 µL of ethyl acetate and 50 µL of the sample was injected into a C18 column (Thermo Fisher Scientific), in an isocratic volume of the mobile phase 50A:50B (A: trifluoroacetic acid (0.05%) in water; B: acetonitrile), running a spectrum from 250 nm to 650 nm with runs of 1 h for all compounds. Silica-TLC of the ethyl acetate extract was also performed to evaluate the presence of decomposition products of the molecules under study. The mobile phase used for these TLCs was *n*-hexane: ethyl acetate (7:3).

### 3.11. Trypanosomicidal Activity In Vitro on Epimastigotes of T. cruzi

Cultures of *T. cruzi* epimastigotes, Tulahuen 2 strain or Y strain, were grown at 28 °C in an axenic medium (brain–heart infusion 33 g/L, tryptose 3 g/L, hemin 0.02 g/L, D-(+)-glucose 0.3 g/L, streptomycin 0.2 g/L, penicillin 200,000 U/L, supplemented with 10 % fetal bovine serum). All the cultures, as well as all the tests, are carried out under aerobic conditions. Parasites in the exponential growth phase were used (cultures of 5–7 days of growth are used, starting at day 0 with 5 million parasites /mL). A suspension of parasites was prepared at a concentration of 4 million cells/mL and 0.6 mL/well was inoculated into a 24-wells plate. Compounds to be evaluated were prepared in a stock solution of 24 mM in DMSO and immediately added to each well to give decreasing concentrations, for example, 25 μM, 10 μM, 5 μM, and 1 μM. The parasites were incubated with the compounds at 28 °C for 5 days. The growth of the parasites was followed by measuring the increase in absorbance at 610 nm, which is proportional to the number of cells. The percentage of inhibition of parasite growth was calculated as PI = {1 − [(Ap-A0p)/(Ac-A0c)]} × 100 when Ap is the absorbance at 610 nm of the culture with treatment at day 5; A0p is the absorbance at 610 nm of the culture treated at day 0; Ac is the absorbance at 610 nm of the culture without treatment (negative control, only up to 0.4% DMSO) at day 5; A0c is the absorbance at 610 nm of the culture without treatment at day 0. The IC_50_ corresponds to the concentration of the compound capable of causing 50% growth inhibition. This is determined by plotting the % inhibition against the log_10_ of the concentration and fitting the points to a sigmoid Boltzmann curve (dose–response curve) using OriginLab 8.5. All the obtained results are the average of at least three independent experiments.

### 3.12. Assay for the Combination of Compounds and Construction of the Isobolograms

To calculate the effects of the different combinations tested in vitro on the epimastigotes form of *T. cruzi*, Tulahuen 2 strain, the method described above was applied. The different concentrations used for each combination of compounds were: 0.25 times IC_50_, 0.5 times IC_50_, 0.75 times IC_50_ and 1-time IC_50_. After five days, the PI was calculated for each mixture as described above. Then, the combination values (CV) were determined graphically and each fraction of the concentration inhibition (FIC) was calculated according to Hallander et al. [62], as the combined IC_50_ divided by the IC_50_ in monotherapy, while CV was defined as the concentration of the compound in the combination that allowed 50 % of inhibition. The inhibitory fraction index was calculated as follows: FICI = (IC_50_ of compound A in combination/IC_50_ of compound A in monotherapy) + (IC_50_ of compound B in combination/IC_50_ of compound B in monotherapy). A FICI value less than 1 indicates synergism; greater than 1 indicates antagonism and equal to 1 indicates additive. The data were also graphically expressed as isobolograms, representing the concentrations of each compound. Each dose of the compound combination was tested in triplicate and two independent experiments. The free software Combenefit (https://www.cruk.cam.ac.uk/research-groups/jodrell-group/combenefit, access date: on 15 July 2018) [63] was also used for the study of the combination of compounds, as it allows the analysis, advanced visualization, and quantification of combinations of drugs and other agents.

### 3.13. Trypanosomicidal Activity in Trypomastigotes of T. cruzi

The experiment was performed on infected, three-month-old, Balb/c mice. Seven days post-infection, when in a parasitemia peak (>1.0 × 10^6^ parasites /mL), the blood of the living (anesthetized) mouse was extracted from the aorta or eye (1 mL per mouse), and citrate was used as an anticoagulant, then seeded in a 96-well plate with a final volume of 100 μL (90 μL of blood + 10 μL from a stock solution of the compound containing 10% DMSO). It was left at 4 °C, 24 h, then 5 μL of each sample was used to count trypomastigotes using a 40x optical microscope (MO) (50-field method). The plate fill was performed by homogenizing the well before filling each well (very slowly), and it was performed in triplicate or quadruplicate to obtain the IC_50_ values of each compound.

### 3.14. In Vivo Studies in the Acute Model of Chagas Disease in Mice 

The protocol was approved by the Paraguayan ethics committee for animal experimentation (No. IORG0010088). Three-month-old Balb/c mice were infected (day 0) with infected blood from mice at the beginning of the peak of parasitemia (parasitemia greater than 1.0 × 10^6^ p /mL with the CL Brener clone of *T. cruzi*) by intraperitoneal injection (10,000 parasites per mouse). Parasitemia was followed from the fourth-day post-infection until all the mice in the group were positive. Parasitemia measurements were performed by optical microscopy. Once all the mice were positive, the treatment was started. The treatment lasted 15 consecutive days; the compounds were administered orally by intragastric cannula once a day. Parasitemia was monitored weekly. At 30 and 60 days, 200 μL of blood was extracted from the mouse tail for serological tests (ELISA test) to detect antigens of *T. cruzi*. Day 60 was the end of the experiment, and the animals were sacrificed.

Hemoconcentration and counting micromethod: the mice were bled by pricking the tail and taking the blood with capillaries. One capillary per mouse draws 8-18 mm in height of blood. The millimeters were converted to μL by a table previously described and calibrated in the laboratory. The capillaries were centrifuged at 3000× *g* for 40 s. The parasitic load in the capillary was observed by optical microscopy (OM) in the capillary: the red blood cells (GR) were distributed at one end and the supernatant serum at the other end, at the interface is the trypomastigotes. Then, the capillary was cut a few mm on the interface towards the GR part and its content was spread on a slide, covered with a coverslip. Then, the total number of parasites in 50 fields (OM at 40× magnification) was counted. This sum was then multiplied by 1/50 to obtain the average number of trypomastigotes per field, corresponding to the number of trypanosomes per 5 mm^3^. This was then compared to the samples from control animals. The mean and standard deviation were calculated by using OriginPro9 and GraphPad Prism 5. Comparisons of parasite suppression were completed by the analysis of variance (two-way ANOVA as a non-parametric statistical test).

### 3.15. Histopathology of the Heart, Spleen, Intestine, Kidney, and Liver of the Mice at the End of the In Vivo Tests in the Acute Model of Chagas Disease

At day 60, after sacrifice, tissue samples were taken from the heart, spleen, intestine, kidney, and liver and stored in 10 % formaldehyde, followed by dehydration in alcohol and xylol solutions and embedding in paraffin. Each organ was individually embedded in paraffin and cut on a 6 mm microtome assembly. The slides were stained with hematoxylin–eosin and viewed under a Zeiss microscope at 10, 40, and 100× magnification. Photographs were taken at all these magnifications with an Olympus X-785 digital camera (OM Digital Solutions Shinjuku, Tokyo, Japan) attached to the microscope. The visual comparison from each treatment was made by looking at the amastigote nests on the entire tissue preparation of around 100 slides per treatment [64].

## 4. Conclusions

In this study, we described the preclinical study of three molecules (**1019**, **1018,** and **793**) for their efficacy against *T. cruzi*. In vitro and in vivo toxicological profiles demonstrated the safety of these molecules to be used as drug candidates for the treatment of Chagas disease. We could demonstrate that triosephosphate isomerase is one of the molecular targets of compounds **793** and **1019**. We have also explored the in vitro metabolic stability of these molecules: two of them, **793** and **1019,** have a short half-life while **1018** was stable in the experimental conditions. Finally, we found synergic in vitro and in vivo combinations between some of the studied molecules. Two combinations, **793** plus **1018** and **793** plus **1019** demonstrated a better behavior than the reference drug Benznidazole in the in vivo model of Chagas disease. Because these molecules are low-cost production compounds, this makes them excellent drug candidates for the treatment of Chagas disease.

## Figures and Tables

**Table 1:** Toxicology profile of compounds **1018**, **1019,** and **793**.

HITS				
Negative Control	Positive Control	1018	1019	793
		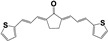	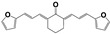	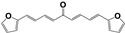
**Toxicological profile**				
**Ames test (mutagenicity)**				
NO	YES (one control per strain)	NO	NO	NO
**Micronucleus test in mice (Genotoxicity) (mean of MnPE ^+^ ±SD ^+^)**				
5 ± 1	36 ± 2 (cyclophosphamide)	5 ± 1	6 ± 2	4 ± 1
Teratogenicity in zebrafish (LD_50_ ± SD) *				
NA	2.4 mM (caffeine)	>25 µM	100 ± 12 µM	22 ± 5 µM
**Acute oral toxicity in mice (Up and Down test, LD_50_, mg/kg of body weight)**				
NA	NA	>2000	>2000	2000

* Doses lethal for 50% of the animals. ^+^ Multimicronucleated cells per mouse. NA not applicable.

**Table 2:** Signals used in ^1^H-NMR experiments to identify the metabolites excreted in the culture medium by epimastigotes of *T. cruzi*, strain Y, and results for compound **1019**.

	Glycine (Gly)	Succinate (Succ)	Pyruvate (Pyr)	Acetate (Ace)	Alanine (Ala)	Lactate (Lac)	Ethanol
δ ^a^ (ppm)	3.547	2.391	2.358	2.121	1.465	1.316	1.170
Multiplicity	S ^b^	S	S	S	D ^c^	d	T ^d^
Integration range	3.55–3.54	2.40–2.38	2.37–2.35	2.12–2.12	1.49–1.44	1.34–1.30	1.20–1.14
*J*(Hz) ^e^	- ^c^	-	-	-	7.24	6.85	7.08
Integration for **1019**	1.53 ± 0.01 *	7.08 ± 0.04 *	6.3 ± 0.2	13.56 ± 0.03	10.3 ± 1.8	4.98 ± 0.03	nq ^f^
Integration of the baseline	1.39 ± 0.03	5.24 ± 0.43	4.01 ± 0.94	11.5 ± 1.2	8.09 ± 0.40	3.99 ± 0.1	nq

^a^ The values of δ (chemical shift) show an error of ± 0.002. ^b^ Singlet. ^c^ Doublet. ^d^ Triplet. ^e^ The values of J (coupling constant) show an error of ± 0.03. * Significant differences in the Student T-test concerning untreated parasites (baseline), *p* values< 0.001. ^f^ not quantified.

**Table 3:** Changes in the ^1^H-NMR experiments, integration ratios of CH_2_/CH_3_ and choline signal, for the different treatments of *T. cruzi* with studied compounds and the control (untreated parasites).

Condition	CH_2_/CH_3_ Ratio	Apparition of the Signal of Choline (3.10–3.30) ppm
**Control**	0.27	YES
**Nfx**	0.25	NO
**Bnz**	0.50	YES
**793**	3.00 *	NO
**1019**	0.18	NO
**1018**	0.06	YES

* Significant differences in the Student *t*-test, *p* values < 0.001.

**Table 4:** Isobolographic analysis of the tested combinations. The optimal molar proportion was derived from the isobolagraphic analysis.

Compound Combination	Effects	FICI	Optimal Molar Proportion
**793** plus **1018**	SYNERGISM	0.5	125/1
**793** plus **1019**	SYNERGISM	0.75	8/1
**793** plus **314**	SYNERGISM	0.75	1/1.2
**793** plus **Bnz**	SYNERGISM	0.5	1/1
**1018** plus **314**	SYNERGISM	0.75	1/133
**1018** plus **1019**	ANTAGONISM	2	-
**1018** plus **Bnz**	ANTAGONISM	2	-
**1260** plus **793**	ANTAGONISM	2	-
**1260** plus **1019**	ANTAGONISM	2	-
**1260** plus **1018**	ANTAGONISM	1.5	-
**Bnz** plus **314**	ADDITION	1	1/2

**Table 5:** Summary of the in vivo trials. It is showing the most promising results regarding the evaluation of compounds in monotherapy and combinations.

Treatment	Doses (µmoles/kg/day)	Reduction in the Parasitemia Peak (%)	Days Post-Infection of the First Parasitemia Peak	Survival (%)
Control	-	0	21	50–100
**793** [25]	192	50	22	83
**1019** [25]	384	75	25	100
	192	60	29	100
**1019** plus **793**	17 + 282 (1/16)	65	15	100
**1018** [25]	192	40	22	40
	1.5	80	24	100
**1018** plus **793**	1.5 + 192 (1/128)	90	19	100
	48 + 48 (1/1)	50	30	88
**Bnz**	48	95	43	100
	38	90	15	100
	10	80	15	100
**Bnz** plus **793**	38 + 192	83	30	100
**314**	102	77	20	100
**314** plus **793**	132 + 192 (1/1.4)	68	26	88

## Data Availability

Data is contained within the article and Supplementary Material.

## Supplementary Materials

The following supporting information can be downloaded at: https://www.mdpi.com/article/10.3390/ph16010020/s1, Table S1: Activity background. The anti-parasitic activity in vitro and in vivo and toxicological profile of the best hits selected from our in-house chemical collection [I–VIII]. Figure S1: In vivo monotherapy activity of selected molecules, Table S2: Ames test using *Salmonella thyphimunum* strain TA98, TA100, TA102, TA1535, TA1537 in the absence of metabolic activation for compound **793**, **1018** and **1019**. Section S1. Preparation details of the compounds. Section S2. Pan assay interference compound virtual check. Section S3. Docking and molecular dynamic figures with structural secondary details. Section S4. 1HNMR spectrum from the metabolomics analysis of the internal metabolites, showing the lactate accumulation. Section S5. Chromatograms from the in vitro metabolism analysis. Section S6. Study of the mechanism of death by flow cytometry for the best combination [65, 66].
