# Supplementary material for: Preclinical Studies and Drug Combination of Low-Cost Molecules for Chagas Disease
Source: Pharmaceuticals (Basel). 2022 Dec 23;16(1):20. doi: 10.3390/ph16010020 (PMC9863266; doi:10.3390/ph16010020)
Supplement: Supplementary file 1 [file pharmaceuticals-16-00020-s001.zip › pharmaceuticals-1961489-supplementary.pdf]

Supporting material

Table S1. Activity background. The anti-trypanosomatid activity identified inhibition in *T. cruzi* bio-system and toxicological profile of the best hits selected from our in-house chemical collection[33–35,44].

**HITS**					
**314**[a]	**266**	**1260**	**1018**	**1019**	**793**
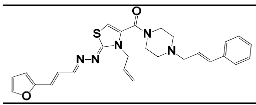	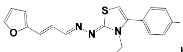	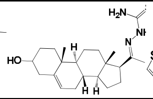	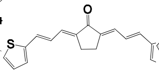	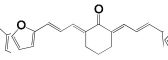	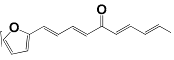
***In vitro* anti-*T. cruzi* activity (multiple strains) (IC_{50})[b]**					
**0.72 µM** amastigotes	**<0.25 µM** amastigotes	**0.1 µM** amastigotes	**40 nM** epimastigotes	**0.6 µM** epimastigotes	**5.0 µM** epimastigotes
***In vitro* anti-*T. brucei* activity (IC_{50})**					
**5.0 µM**	**5.0 µM**	unknown	**15 µM**	**22 µM**	**17 µM**
***In vitro* anti-*Leishmania spp* (cutaneous) activity (IC_{50})**					
**12 µM** promastigotes	**7 µM** promastigotes	**<20 µM** promastigotes	**>100 µM** promastigotes	**7.0 µM** promastigotes	**36 µM** promastigotes
***In vitro* anti-*Leishmania infantum* (visceral) activity (IC_{50})**					
**4.0 µM** promastigotes	**2.0 µM** promastigotes	**<0.2 µM** amastigotes	**11 µM** promastigotes	**13 µM** promastigotes	**31 µM** promastigotes
**Selectivity index >10 (IC_{50} mammalian cell/IC_{50} *parasites*)**					
**Identified *T. cruzi* bio-system inhibited by compounds**					
Cruzipain inhibitor **IC_{50} 4.3 µM**	unknown	Unknown	Triosephosphate isomerase inhibitor **IC_{50} 4.7 µM**	Triosephosphate isomerase inhibitor **IC_{50} 86 nM**	Triosephosphate isomerase inhibitor **IC_{50} 3.0 µM**
***In vitro* stability (microsomal, plasma, other solutions)**					
**high**	**high**	**High**	Unknown	unknown	unknown
**Toxicology and efficacy**					
**Ames test (mutagenicity)**					
**NO**	**NO**	**NO**	Unknown	unknown	**NO**
**Micronucleus test in mice (Genotoxicity)**					
**NO**	**NO**	**NO**	Unknown	unknown	unknown
**Teratogenicity in zebrafish (LD_{50}, µM)[c]**					
**>25**	**>50**	**>15**	Unknown	unknown	unknown
**Acute oral toxicity in mice (Up and Down test, LD_{50}, mg/kg of body weight)**					
**>2000**	**>2000**	**>2000**	Unknown	unknown	unknown
**Full control of the parasitemia *in vivo* at 50 mg/kg in the murine model of Chagas disease and cutaneous model of Leishmaniasis**					
a-The numbers are referred to the chemical collection. b- Is the concentration that inhibited the 50 of the biological response. c- the dose that kills 50% of the animals					

Figure S1. *In vivo* monotherapy activity of selected molecules in the Chagas disease murine model. The black bar showed 50 % of the parasitemia pick at day 25 post-infection. The doses, in µmoles/kg/day for 14 days, are shown in parenthesis at the side of the compound code[33–35,44].

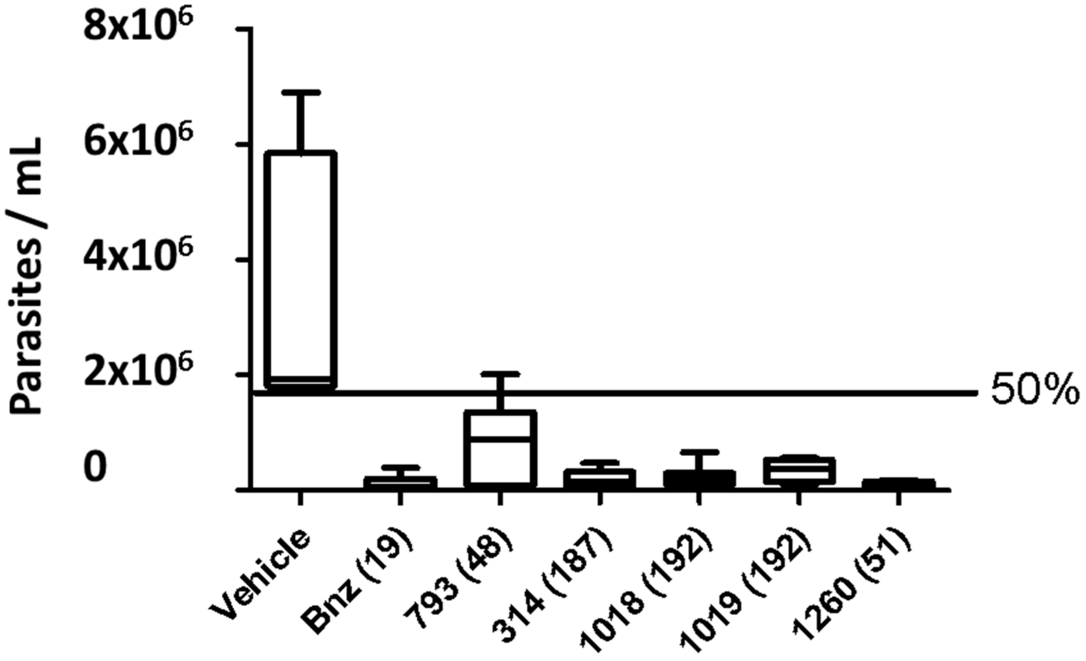

1: Preparation details of the compounds

Compounds Characterizations. All of the synthesized compounds were chemically characterized by thin layer chromatography (TLC), nuclear magnetic resonance (^{1}H NMR, ^{13}C NMR), and elemental microanalyses (CHN). Alugram SIL G/UV254 (Layer: 0.2 mm) (Macherey-Nagel GmbH & Co. KG., Düren, Germany) was used for TLC, and silica gel 60 (0.040−0.063 mm, Merck) was used for flash column chromatography. The NMR spectra were recorded on a Bruker DPX 400 (400 MHz for ^{1}H and 100 MHz for ^{13}C), using TMS as the internal standard and with the indicated deuterated solvent; the chemical shifts are reported in ppm (δ) and coupling constants (J) values are given in Hertz (Hz). Signal multiplicities are represented by s (singlet), d (doublet), dd (double doblet), t (triplet), tt (triple triplet), and m (multiplet). Structural assignments were corroborated by HMBC and HSQC experiments. Mass spectrometry experiments were performed on a HEWLETT PACKARD MSD 5973 or a LC/MSD-Serie 100 using electronic impact (EI) or electrospray ionization (ESI), respectively. To determine the purity of the compounds, elemental microanalyses obtained on a Carlo Erba Model EA1108 elemental analyzer from vacuum-dried samples were used. The analytical results for C, H, and N were within ±0.4 of the theoretical values. Melting points were recorded on ELECTROTHERMAL IA-9100 equipment, and they were not corrected. Samples of all product of this work are available from the authors.

Preparation of the compounds **793**, **1018** and **1019**

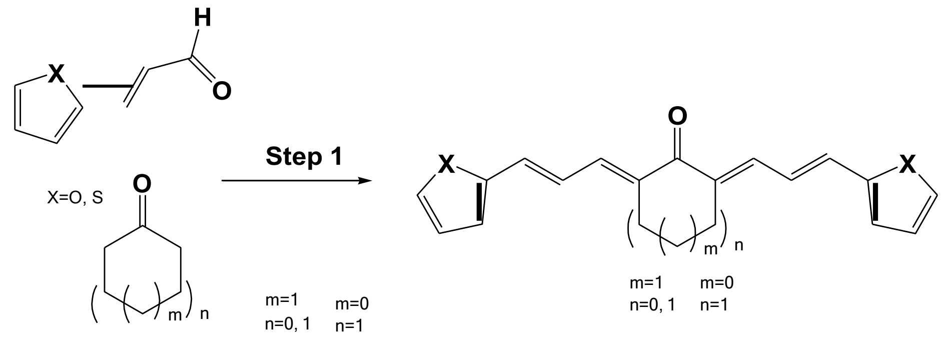

A mixture of the ketone reactant (2.2 mmol) and the corresponding aldehyde (4.5 mmol) were dissolved in 4.4 mL of water and 3.5 mL of ethanol in a 50 mL flask equipped with a magnetic stirrer. Then NaOH (11.2 mmol) was added and the reaction mixture was stirred for 24 h at room temperature. The reaction was monitored using TLC with silica as stationary phase and a mixture of hexane:ethyl acetate (7:3) as the mobile phase. The precipitated solid was filtered under vacuum, washed with water and recrystallized from ethanol.

(1*E*,3*E*,6*E*,8*E*)-1,9-Di(furan-2-yl)nona-1,3,6,8-tetraen-5-one (**793**): orange solid, yield =98%, m.p.= 121-124 °C, ^{1}H-NMR ($CDCl_3$) δ (ppm): 7.46 (2H, d, J=1.6, H15,18), 7.42 (2H, d, J=11.0, H3,8), 6.87 (2H, d, J=11.1, H4,9), 6.75 (2H, d, J=11.1, H6,11), 6.55 (2H, d, J=1.6, H14,19), 6.52 (2H, d, J=11.0, H5,10), 6.47 (2H, d, J=1.8, H13,20). ^{13}C-

NMR ($CDCl_3$) δ (ppm): 100 (C13,20), 113 (C14,19), 125 (C6,11) ,128 (C3,8), 143 (C4,9), 153 (C15,18), 189 (C1). MS (EI) m/z (%): 266 ($M^{+•}$, 100); 185 ($C_{12}H_{10}O_2^+$, 18.49); 81 ($C_5H_4O^+$, 38.62). Elemental Analysis: C, 76.68; H, 5.30; O, 18.02

(2*E*,6*E*)-2,6-Bis[(*E*)-3-(furan-2-yl)allylydene]cyclohexanone (**1018**): brown solid, yield =60%, m.p.= 156-158 °C, ^{1}H-NMR ($CDCl_3$) δ (ppm): 7.65 (2H, d, J=6.8, H18,21), 7.35 (2H, s, H7,8), 7.05 (2H,d, J=12.2, H9,12), 6.75 (2H, d, J=12.2, H10,13), 6.55 (2H, m, H16,23), 6.41 (2H, m, H17,22), 2.79 (4H, m, H2,4), 1.88 (2H, m, H3). ^{13}C-NMR ($CDCl_3$) δ (ppm): 193 (C6), 149 (C11,14), 145 (C18,21), 142 (C1,5), 137 (C7,8), 130 (C9,12), 129 (C10,13), 113 (C17,22), 111 (C16,23), 28 (C2,4), 24 (C3). MS (EI) m/z (%): 306($M^{+•}$, 100); 184($C_{13}H_{12}O_4^+$; 10). Elemental Analysis: C, 78.41; H, 5.92; O, 15.67.

(2*E*,5*E*)-2,5-Bis[(*E*)-3-(thien-2-yl)allylydene]cyclopentanone (**1019**): orange solid, yield =100%, m.p.= 168-170 °C, ^{1}H-NMR ($CDCl_3$) δ (ppm): 7.32 (2H, d, J=5.0, H17,20), 7.49 (2H, s, H6,7), 7.20 (2H, d, J=3.5, H15,22), 7.15 (2H, d, J=4.5, H16,21), 7.11 (2H, d, J=15.3, H8,10), 6.78 (2H, d, J=15, H9,11), 2.90 (2H, s, H2,3). ^{13}C-NMR ($CDCl_3$) δ (ppm): 198 (C5), 141 (C12,13), 140 (C1,4), 132 (C6,7), 130 (C9,11), 129 (C8,10), 128 (C17,20), 127 (C16,21), 126 (C15,22), 30 (C2,3). MS (EI) m/z (%): 324.06 ($M^{+•}$, 100). Elemental Analysis: C, 70.33; H, 4.97; O, 4.93; S, 19.77.

Preparation of **266** and **314**

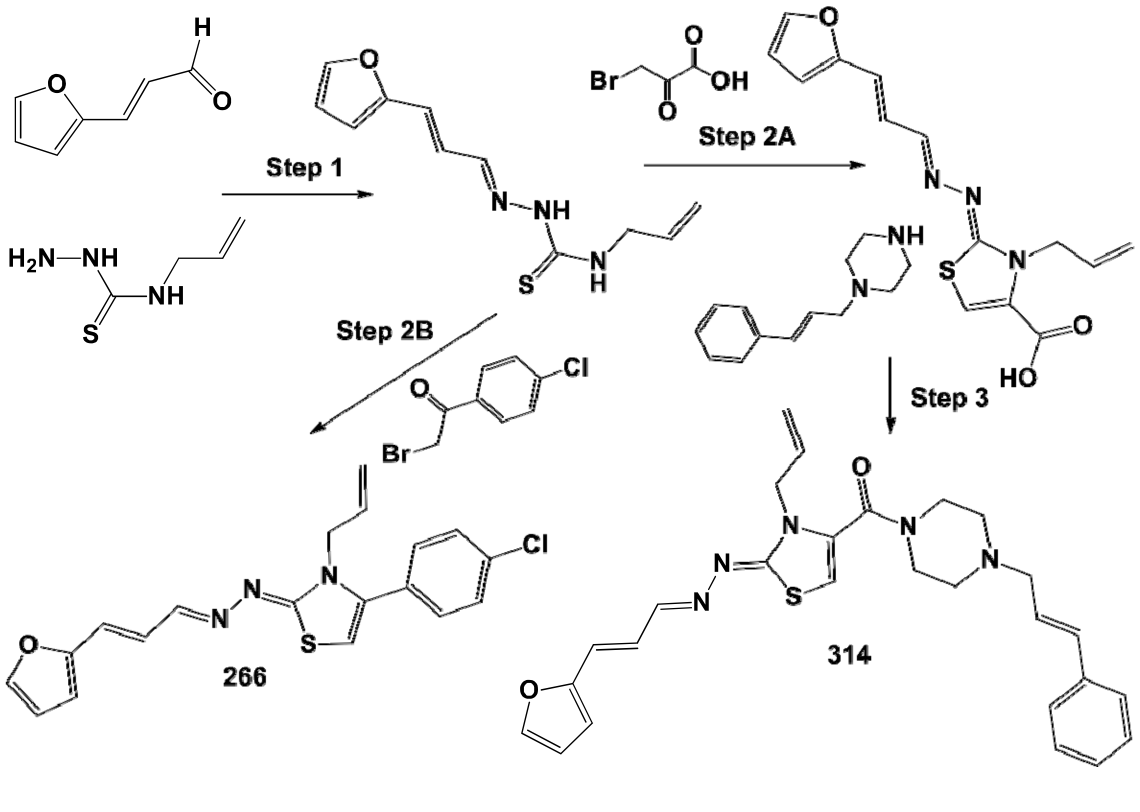

Scheme S2. Synthetic steps for the preparation of **314** and **266**.

Step 1. A mixture of the corresponding aldehyde (1.05 equiv.), the corresponding thiosemicarbazide (1.00 equiv.), catalytic amount of *p*-toluenesulfonic acid, and dry

toluene (1 mL per 100 mg of aldehyde) was stirred at room temperature until disappearance of the aldehyde (12−24 h, checked by TLC, SiO_2, using as a mobile phase petroleum ether:ethyl acetate (70:30)). After that, the precipitate was filtered off and washed with petroleum ether. The solid was crystallized from ethanol.

Step 2. A mixture of the corresponding thiosemicarbazone (1.0 equiv.), the corresponding α-haloketone (1.2 equiv.), and ethanol 98% (1 mL per 100 mg of thiosemicarbazone) was heated at reflux until disappearance of the thiosemicarbazone (4−10 h, checked by TLC, SiO_2, and petroleum ether:ethyl acetate (70:30)). After that, the mixture was cooled at room temperature, and the precipitate was filtered off and washed with ethanol:water (80:20). The solid was crystallized from ethanol or ethanol:water.

1Z-[3-Allyl-4-(4-chlorophenyl)thiazol-2(3H)-ylidene]-2E-[3-(2-furyl)-2E-propenylidene] hydrazine hydrobromide hydrate, (**266**). Orange solid, 97 %; mp: 200 ºC (d), ^{1}HNMR ($CDCl_3$) δ (ppm): 4.71 (2H s), 5.05 (2H d 17), 6.76 (1H d *J*16), 6.47 (m, 2H); 6.89 (1H dd 16/5.8), 7.36 (2H d 8), 7.44 (2H d 8), 7.46 (1H bs), 8.48 (1H bs), 8.25 (1H s). ^{13}C NMR ($CDCl_3$): δ 48, 117, 103, 111, 112, 123, 126, 129, 130, 131, 136, 140, 144, 152, 154, 168. MS (EI) m/z (abundance, %): 369 (M+., 100), 235 (42); UV: λmax: 379 nm, ε = 14.7 ± 0.5 cm-1 mM-1. Found: C, 49.0; H, 3.9; N, 9.1; S, 6.9. C19H19BrClN3O2S Exact Mass: 467.01 C, 48.68; H, 4.09; Br, 17.04; Cl, 7.56; N, 8.96; O, 6.83; S, 6.84.

Step 3. A mixture of the corresponding acid (1.0 equiv.), thionyl chloride (1.2 equiv.) and dry toluene (1 mL per 100 mg of acid) was heated at 100 ºC for 1 h. After that, a solution of the corresponding amine (1.0 equiv.) and triethylamine (5.0 equiv.) were added dropwise during 30 min to the reaction cooled at 0 ºC. The mixture of reaction was stirred at room temperature until disappearance of the activated acid (12-24 h, checked by TLC, Al_2O_3, petroleum ether:ethyl acetate (70:30)). After that, the solvent was evaporated *in vacuo* and the residue was partitioned between methylene dichloride and saturated aqueous solution of sodium bicarbonate. The organic layer was washed with aqueous phosphate buffer (pH 4-5), dried with anhydrous sodium sulfate and evaporated *in vacuo*. The desired product was purified, from the residue of evaporation, by column chromatography (Al_2O_3, petroleum ether:ethyl acetate (0 to 40 %)).

(2*E*,2*Z*)-3-Allyl-4-[((*E*)-4-cinnamylpiperazin-1-yl)carbonyl]-2-[2-((*E*)-3-(furan-2-yl)propenylydene)hydrazono]-2,3-dihydrothiazole (**314**): yellow solid, yield =60 %, m.p.= 157-159 ºC. ^{1}HNMR ($CDCl_3$) δ (ppm): 2.52 (bs, 4H), 3.2 (d, J= 6.6 Hz, 2H), 3.68 (bs, 4H), 4.66 (d, J= 5.6 Hz, 2H), 5.19 (m, 2H), 5.91 (m, 1H), 6.15 (s,1H), 6.25 (m, 1H), 6.46 (m, 1H), 6.56 d, J=16Hz, 1H), 6.64 (d, J=16, 1H), 6.92 (dd, J=9.9/16, 1H), 7.38 (m, 4H), 7.44 (m, 1H), 8.07 (d, J=9.9, 1H). ^{13}C NMR ($CDCl_3$): δ 142, 110,132, 125, 152, 168, 104, 142, 170, 42, 47, 60, 124, 134, 128, 118. MS (EI) m/z (abundance, %): 487 ($M^{+\cdot}$, 13), 407 (10), 117 (100). UV: 387 nm (ε = 27.2 ± 0.4 $cm^{-1}mM^{-1}$). Found: C, 66.6; H, 6.0; N, 14.1; S, 6.4.

Preparation of **1260**

Step 1. A mixture of pregnolone (1.05 equiv.), thiosemicarbazide (1.00 equiv.), catalytic amount of *p*-toluenesulfonic acid, and dry toluene (1 mL per 100 mg of ketone) was stirred at room temperature until disappearance of the aldehyde (12−24 h, checked by TLC, SiO_2, using as a mobile phase petroleum ether:ethyl acetate (70:30)). After that, the precipitate was filtered off and washed with petroleum ether. The solid was crystallized from ethanol.

1-(3β-Hydroxy-pregn-5-ene-20*E*-ylydene)thiosemicarbazide (**1260**): White powder, yield: 95%, white powder, m.p. 245–248 °C; ^{1}H RMN ($CDCl_3$) δ (ppm): 0.63 (s, 3H), 0.99 (s, 3H), 2.83 (t, 1H, J = 8.5 Hz), 3.52 (m, 1H), 5.35 (t, 1H, J = 2.5 Hz), 8.56 (s, 2H). ^{13}C RMN ($CDCl_3$) δ (ppm): 13.2, 19.5, 21.2, 23.5, 25.2, 31.4, 32.6, 32.9, 37.2, 37.8, 39.5, 42.5, 45.5, 50.7, 57.5, 62.4, 71.2, 121.4, 165.4, 181.2 $C_{22}H_{35}N_3OS$. ESI-MS (m/z): 389.25 (100.0%), 390.25 (25.7%), 391.25 (10.0%), 392.25 (1.1%). Elemental analysis: C, 67.82; H, 9.05; N, 10.79; O, 4.11; S, 8.23.

2: Pan assay interference compound: Virtual check

Interference Compounds check. The active compounds (**793**, **1019**, **1018**, **314** and **1260**) were tested online fallowing the recommendation on the Ecstasy and Agony of Assay Interference Compoundsi. The screenshot for the compounds is showed above, was made in four different on line software (http://zinc15.docking.org/patterns/home, http://www.cbligand.org/PAINS/, https://fafdrugs4.rpbs.univ-paris-diderot.fr/links, http://advisor.docking.org), for PAINS and aggregator compounds. Any of them show problem in those test. Also these compound were used in many other enzymatic assay, and were not active, demonstration their non-interference status.ii,iii,iv,v

Compound **793**:

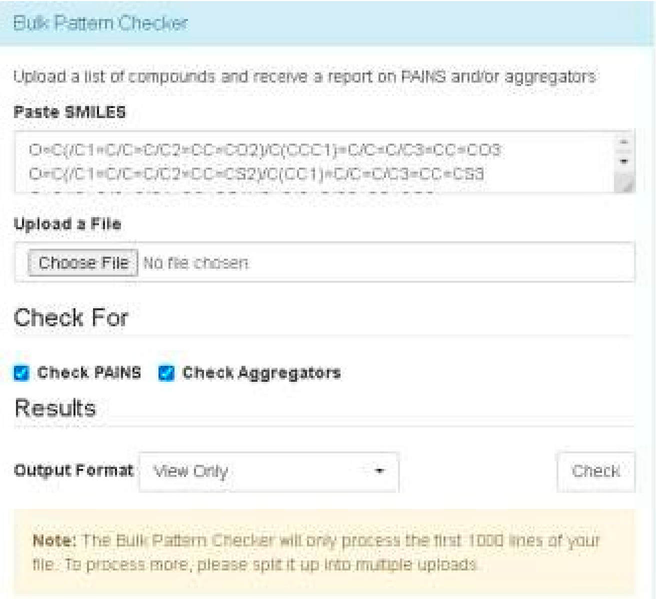

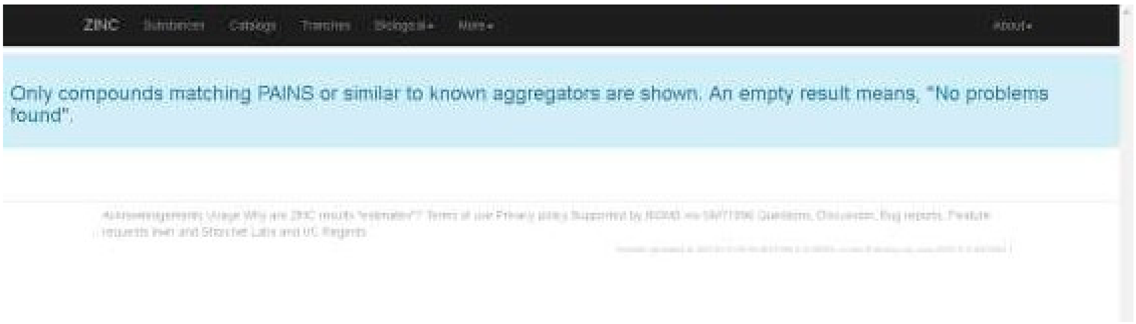

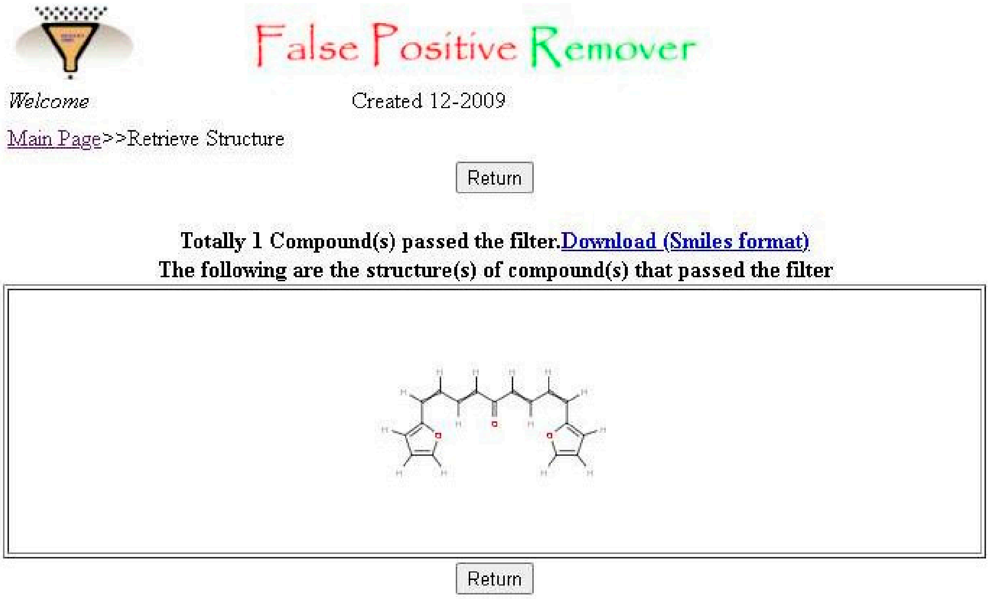

1

FAFDrugs4 Results

Downloads

- **Accepted Molecules:** [SDF]
- **Protonated Accepted Molecules (pH7.4):** [SDF]
- **Intermediate Molecules:** [SDF]
- **Protonated Intermediate Molecules (pH7.4):** [SDF]
- **Rejected Molecules:** [SDF]
- **Protonated Rejected Molecules:** [SDF]
- **Duplicates:** *None*
- **Mixtures:** *None*
- **Empty Structures:** *None*
- **Isotopes and Inorganics:** *None*
- **Large Compounds:** *None*
- **PAINS:** [SDF]
- **Covalent Inhibitors:** *None*
- **Descriptors:** [CSV]
- **Groups:** *None*
- **PAINS:** [CSV]

Filtering statistics

- **Input molecules:** 1
- **Duplicates:** 0
- **Mixtures:** 0
- **Large Compounds:** 0
- **Isotopes and Inorganics:** 0
- **Empty Structures:** 0
-

  Filtered molecules: 1
- **Rejected molecules:** 0
- **Accepted molecules:** 1
- **Intermediate molecules:** 0
- **PAINS (Pan Assays Interferences Compounds):** 0
- **Covalent Inhibitors:** 0

Main PhysChem Descriptors Analysis

Click on a plot to enlarge the picture

Your bank contains less than 30 molecules, thus histograms were not generated...

Library of compounds computed descriptors:

Click on the compound ID to to get detailed compound results.

ID	MW	logP	logD	logSw	tPSA	RotatableBonds	RigidBonds	Flexibility	HBDonors	HBAcceptors	HBD_HBA	Rings	MaxSizeRing	Charges	TotalCharge	HeavyAtoms	CarbonAtoms	HeteroAtoms	RatioH/C	LipinskiViolations	Solubility (mg/l)	SolubilityForecastIndex	OralBioavailability (VEBER)	OralBioavailability (EGAN)	TrafficLights	4_400	3_75	Phospholipidosis	Fsp3	StereoCenters	PPI_Friendly	Status

Loading data from server

ID	MW	logP	logD	logSw	tPSA	RotatableBonds	RigidBonds	Flexibility	HBDonors	HBAcceptors	HBD_HBA	Rings	MaxSizeRing	Charges	TotalCharge	HeavyAtoms	CarbonAtoms	HeteroAtoms	RatioH/C	LipinskiViolations	Solubility (mg/l)	SolubilityForecastIndex	OralBioavailability (VEBER)	OralBioavailability (EGAN)	TrafficLights	4_400	3_75	Phospholipidosis	Fsp3	StereoCenters	PPI_Friendly	Status

---

Filtering input parameters:

Your input parameters correspond to the **nophyschem** parameters (see the documentation).

Click on a parameter to get related informations in the official FAF-Drugs4 documentation.

max_lipinski	max_ring	inf_r	sup_r
inf_mw	sup_mw	inf_hb	sup_hb
inf_hbd	sup_hbd	inf_hba	sup_hba
inf_hva	sup_hva	inf_nc	sup_nc
inf_nbb	sup_nbb	inf_nrb	sup_nrb
inf_psa	sup_psa	inf_logp	sup_logp
inf_cf	sup_cf	inf_sc	sup_sc
inf_c	sup_c	inf_h	sup_h
inf_ch	sup_ch		

Compound **1018**:

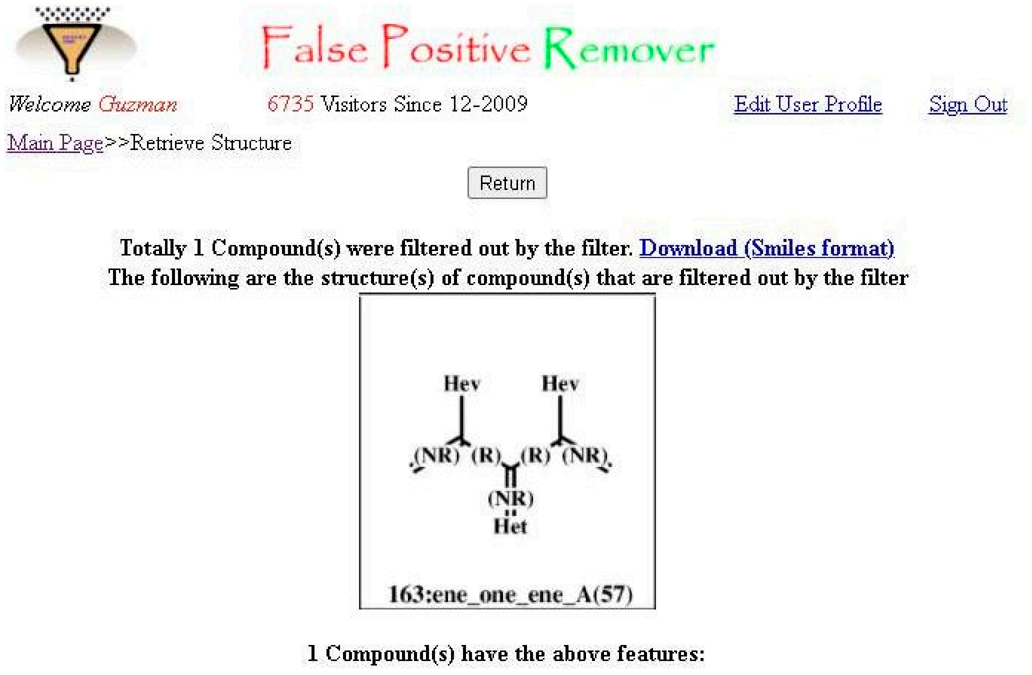

1 Compound(s) have the above features:

C=[!R]C(Hev)-[R]C(=[!R]Het)-[R]C(=[!R]C)Hev

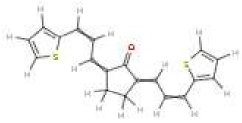

Return

1

FAFDrugs4 Results

Downloads

- **Accepted Molecules:** [SDF]
- **Protonated Accepted Molecules (pH7.4):** [SDF]
- **Intermediate Molecules:** [SDF]
- **Protonated Intermediate Molecules (pH7.4):** [SDF]
- **Rejected Molecules:** [SDF]
- **Protonated Rejected Molecules:** [SDF]
- **Duplicates:** *None*
- **Mixtures:** *None*
- **Empty Structures:** *None*
- **Isotopes and Inorganics:** *None*
- **Large Compounds:** *None*
- **PAINS:** [SDF]
- **Covalent Inhibitors:** *None*
- **Descriptors:** [CSV]
- **Groups:** *None*
- **PAINS:** [CSV]

Filtering statistics

- **Input molecules:** 1
- **Duplicates:** 0
- **Mixtures:** 0
- **Large Compounds:** 0
- **Isotopes and Inorganics:** 0
- **Empty Structures:** 0
- **Filtered molecules:** 1
- **Rejected molecules:** 0
- **Accepted molecules:** 0
- **Intermediate molecules:** 1
- **PAINS (Pan Assays Interferences Compounds):** 1
- **Covalent Inhibitors:** 0

Main PhysChem Descriptors Analysis

Click on a plot to enlarge the picture

Your bank contains less than 30 molecules, thus histograms were not generated...

Library of compounds computed descriptors:

ID	MW	logP	logD	logSw	tPSA	RotatableBonds	RigidBonds	Flexibility	HBDonors	HBAcceptors	HBD_HBA	Rings	MaxSizeRing	Charge	TotalCharge	HeavyAtoms	CarbonAtoms	HeteroAtoms	RatioH/C	LipinskiViolations	Solubility (mg/l)	SolubilityForecastIndex	Oral Bioavailability (VEBER)	Oral Bioavailability (EGAN)	TrafficLights	4_400	3_75	Phospholipidosis	Fsp3	StereoCenters	PPI_Friendly	Status

Loading data from server

ID	MW	logP	logD	logSw	tPSA	RotatableBonds	RigidBonds	Flexibility	HBDonors	HBAcceptors	HBD_HBA	Rings	MaxSizeRing	Charge	TotalCharge	HeavyAtoms	CarbonAtoms	HeteroAtoms	RatioH/C	LipinskiViolations	Solubility (mg/l)	SolubilityForecastIndex	Oral Bioavailability (VEBER)	Oral Bioavailability (EGAN)	TrafficLights	4_400	3_75	Phospholipidosis	Fsp3	StereoCenters	PPI_Friendly	Status

Filtering input parameters:

Your input parameters correspond to the **nophyschem** parameters (see the documentation).

Click on a parameter to get related informations in the official FAF-Drugs4 documentation.

max_lipinski	max_ring	inf_r	sup_r
inf_mw	sup_mw	inf_hb	sup_hb
inf_hbd	sup_hbd	inf_hba	sup_hba
inf_hva	sup_hva	inf_nc	sup_nc
inf_nbb	sup_nbb	inf_nrb	sup_nrb
inf_psa	sup_psa	inf_logp	sup_logp
inf_cf	sup_cf	inf_sc	sup_sc
inf_c	sup_c	inf_h	sup_h

[inf_ch](#) [sup_ch](#)

Compound **1019**:

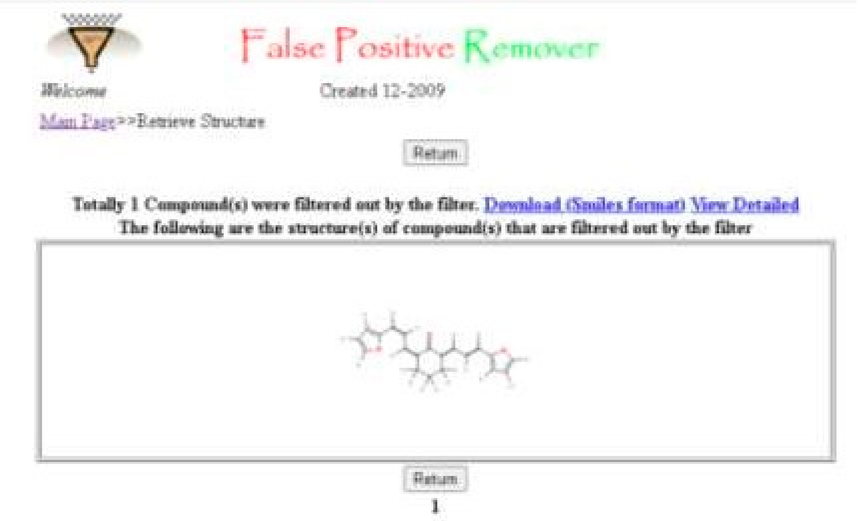

FAFDrugs4 Results

Downloads

- **Accepted Molecules:** [SDF]
- **Protonated Accepted Molecules (pH7.4):** [SDF]
- **Intermediate Molecules:** [SDF]
- **Protonated Intermediate Molecules (pH7.4):** [SDF]
- **Rejected Molecules:** [SDF]
- **Protonated Rejected Molecules:** [SDF]
- **Duplicates:** *None*
- **Mixtures:** *None*
- **Empty Structures:** *None*
- **Isotopes and Inorganics:** *None*
- **Large Compounds:** *None*
- **PAINS:** [SDF]
- **Covalent Inhibitors:** *None*
- **Descriptors:** [CSV]
- **Groups:** *None*
- **PAINS:** [CSV]

Filtering statistics

- **Input molecules:** 1
- **Duplicates:** 0
- **Mixtures:** 0
- **Large Compounds:** 0
- **Isotopes and Inorganics:** 0
- **Empty Structures:** 0
- **Filtered molecules:** 1
- **Rejected molecules:** 0
- **Accepted molecules:** 0
- **Intermediate molecules:** 1
- **PAINS (Pan Assays Interferences Compounds):** 1
- **Covalent Inhibitors:** 0

Main PhysChem Descriptors Analysis

Click on a plot to enlarge the picture

Your bank contains less than 30 molecules, thus histograms were not generated...

Library of compounds computed descriptors:

ID	MW	logP	logD	logSw	tPSA	RotatableBonds	RigidBonds	Flexibility	HBDonors	HBAcceptors	HBD_HBA	Rings	MaxSizeRing	Charge	TotalCharge	HeavyAtoms	CarbonAtoms	HeteroAtoms	RatioH/C	LipinskiViolations	Solubility (mg/l)	SolubilityForecastIndex	Oral Bioavailability (VEBER)	Oral Bioavailability (EGAN)	TrafficLights	4_400	3_75	Phospholipidosis	Fsp3	StereoCenters	PPI_Friendly	Status

Loading data from server

ID	MW	logP	logD	logSw	tPSA	RotatableBonds	RigidBonds	Flexibility	HBDonors	HBAcceptors	HBD_HBA	Rings	MaxSizeRing	Charge	TotalCharge	HeavyAtoms	CarbonAtoms	HeteroAtoms	RatioH/C	LipinskiViolations	Solubility (mg/l)	SolubilityForecastIndex	Oral Bioavailability (VEBER)	Oral Bioavailability (EGAN)	TrafficLights	4_400	3_75	Phospholipidosis	Fsp3	StereoCenters	PPI_Friendly	Status

---

Filtering input parameters:

Your input parameters correspond to the **nophyschem** parameters (see the documentation).

Click on a parameter to get related informations in the official FAF-Drugs4 documentation.

---

max_lipinski	max_ring	inf_r	sup_r
inf_mw	sup_mw	inf_hb	sup_hb
inf_hbd	sup_hbd	inf_hba	sup_hba
inf_hva	sup_hva	inf_nc	sup_nc
inf_nbb	sup_nbb	inf_nrb	sup_nrb
inf_psa	sup_psa	inf_logp	sup_logp
inf_cf	sup_cf	inf_sc	sup_sc

inf_c sup_c inf_h sup_h

inf_ch sup_ch

Table S2. Ames test using *Salmonella thyphimunum* strain TA98, TA100, TA102, TA1535, TA1537 in the absence of metabolic activation for compound 793, 1018 and 1019.

Compound	Doses (µg/plate)	TA98	TA100	TA102	TA1535	TA1537
**793**	0	6±1	109±5	204±8	11±3	31±7
	500	5±1	69±1	222±6	20±1	30 ± 3
	166	3 ± 1	58±3	203±2	18±1	28± 3
	55	3±1	74±6	194±3	14± 3	24± 4
	18	9±3	76±5	176±1	12±1	18±1
	6	5±2	89±3	171±1	8±1	16 ± 1
**1018**	0	6±1	60±5	204±8	11±3	31±7
	800	7±2	125±4	243±1	22±1	40.5±1.5
	266	6±3	113±3	237±1	16±1	36±1
	88	11±2	101±3	231±2	9±1	32±1
	29	25±1	90±3	222 ±2	8±1	28 ±2
	9	4±1	80±1	216±1	3±1	25±3
**1019**	0	15±2	109±5	204±8	11±3	31±7
	400	24 ± 3	81 ± 3	230±2	15±1	60 ± 2
	133	20 ±1	74±6	217±3	17±1	51± 3
	44	19 ± 2	62±11	207±2	14±1	44 ± 1
	14	27 ±7	79±2	201±2	14± 2	36± 3
	4	15 ±1	60±5	187±2	9± 1	29± 1

Supporting material 3. Docking and molecular dynamic figures with structural secondary details. Molecular dynamics and docking studies. Site of interaction of compound **793** in TcTIM after molecular docking (A) and after molecular dynamics (B). C) Compound **793** interaction site at the TcTIM interface. In sky-blue are represented the carbons; hydrogens in blank; in blue the nitrogen and in red the oxygen. D) RMSF of the residues according to the Cα. The greatest shift is observed in the loop6 of monomer B for compound **1019**.

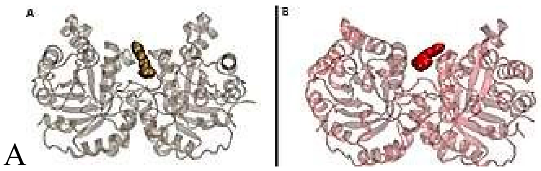

A B

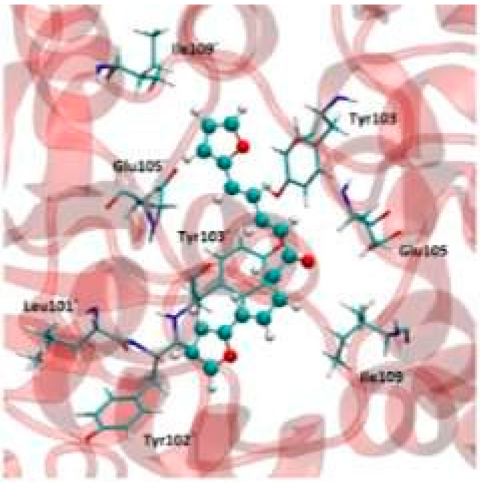

C

D

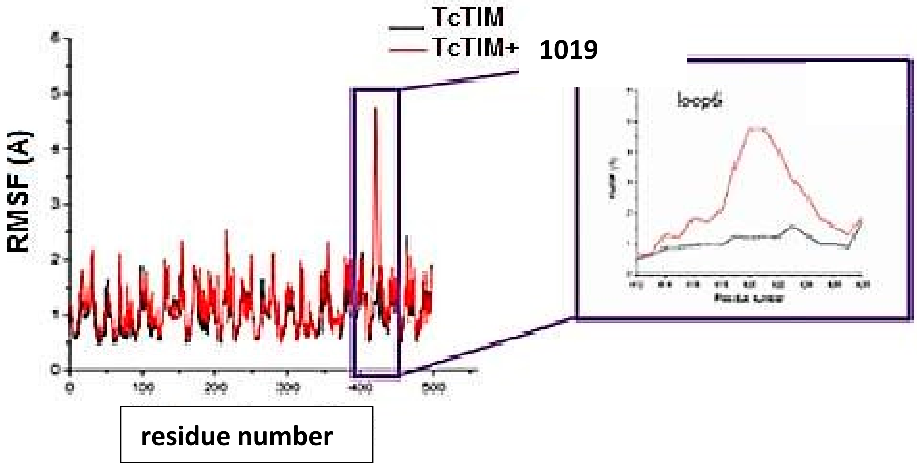

Supporting material 4. ¹HNMR spectrum from the metabolomics analysis showing the lactate accumulation. This spectrum correspond to the internal metabolites under **1019** treatment. In red are the spectrum for the condition with inhibition (with **1019**) and in blue the control parasites, (10 spectrum overlapping).

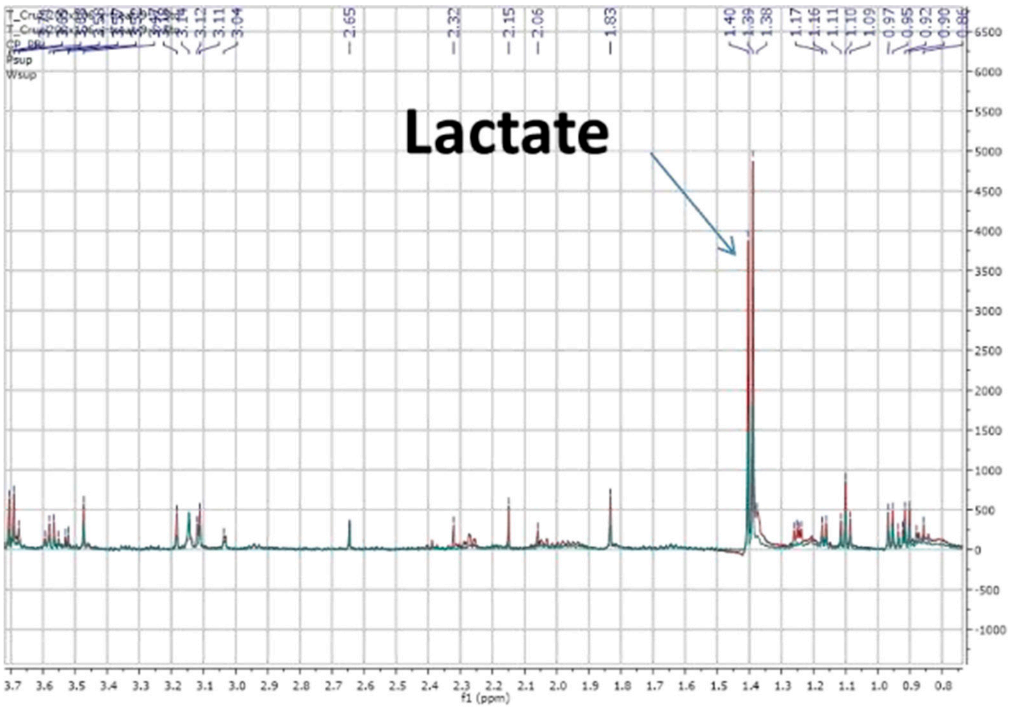

Supporting material 5. Chromatograms from the metabolization analysis. Study of metabolization of compound **793** by HPLC. A) **793** (solution in DMSO at a concentration of 0.4 mM) measured at 440 nm, using as mobile phase water and trifluoroacetic acid (0.05%):acetonitrile (50:50). B) FC + **793** at 0.4 mM (incubation time: 0 min). C) FC + **793** at 0.4 mM (incubation time: 30 minutes). D) FC + **793** at 0.4 mM (incubation time 60 min hour). E) FM + **793** at 0.4 mM (incubation time 0 min). F) FM + **793** at 0.4 mM (incubation time 30 minutes). G) FM + **793** at 0.4 mM (incubation time 60 min).

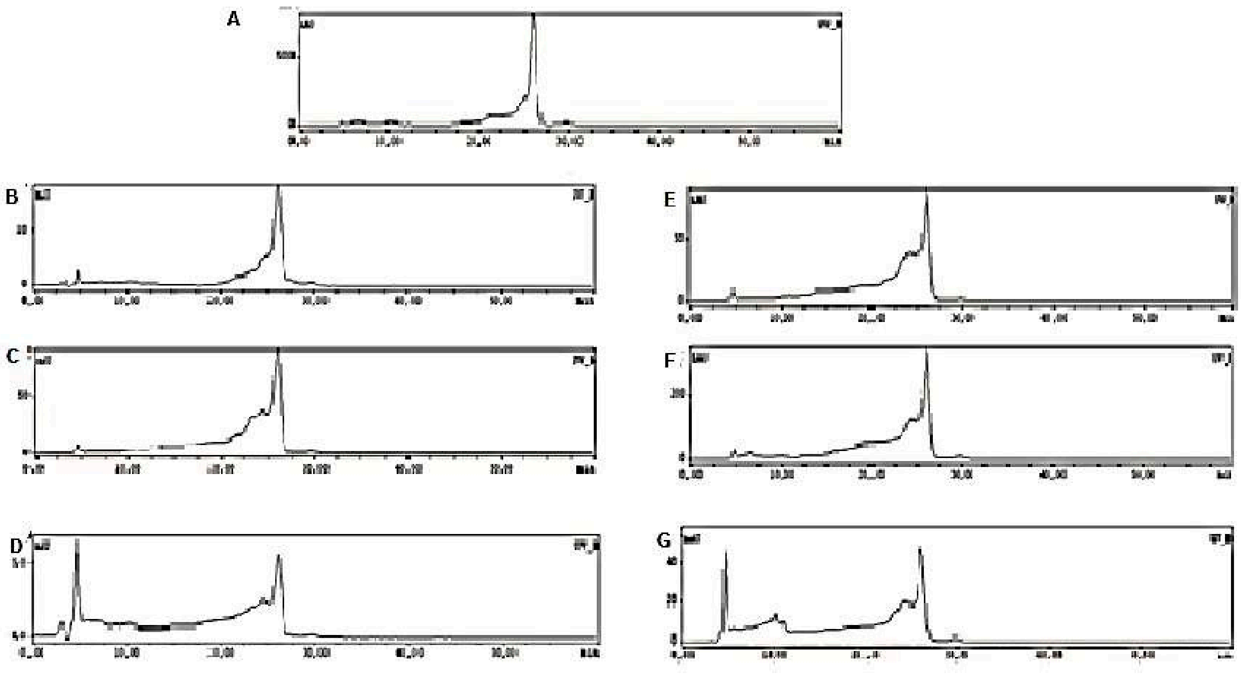

Supporting material 6. Analysis of the viability and mechanism of death by flow cytometry in parasites at 6 and 24 h of incubation at a 20 × IC50 concentration of Nfx, Bnz, 793 (2), 1019 (11), 1018 (16) and 793+1018 (2+16) A) CA-AM/IP labeling. B) AV/IP labeling

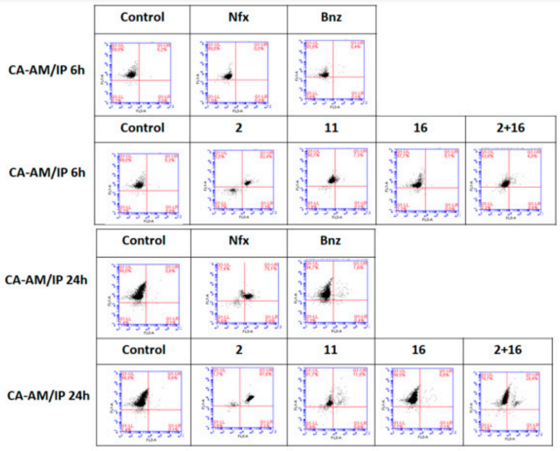

[i] Aldrich C, Bertozzi C, Georg GI, Kiessling L, Lindsley C, Liotta D, Merz KM Jr, Schepartz A, Wang S. The Ecstasy and Agony of Assay Interference Compounds. ACS Med Chem Lett. 2017 Feb 28;8(4):379-382. doi: 10.1021/acsmedchemlett.7b00056. PMID: 28435522; PMCID: PMC5392755.

ii Risi, G., Aguilera, E., Ladós, E., Suárez, G., Carrera, I., Álvarez, G., Salinas, G., 2019. Caenorhabditis elegans Infrared-Based Motility Assay Identified New Hits for Nematicide Drug Development. Vet. Sci. 6, 29. doi:10.3390/vetsci6010029

iii Saramago, L., Gomes, H., Aguilera, E., Cerecetto, H., González, M., Cabrera, M., Alzugaray, M., da Silva Vaz Junior, I., Nunes da Fonseca, R., Aguirre-López, B., Cabrera, N., Pérez-Montfort, R., Merlino, A., Moraes, J., Álvarez, G., 2018. Novel and Selective Rhipicephalus microplus Triosephosphate Isomerase Inhibitors with Acaricidal Activity. Vet. Sci. 5, 74. doi:10.3390/vetsci5030074

iv Ferraro, F., Corvo, I., Bergalli, L., Ilarraz, A., Cabrera, M., Gil, J., Susuki, B.M., Caffrey, C.R., Timson, D.J., Robert, X., Guillon, C., Freire, T., Álvarez, G., 2020. Novel and selective inactivators of Triosephosphate isomerase with anti-trematode activity. Sci. Rep. 10, 1–13. doi:10.1038/s41598-020-59460-y

v Aguilera, E., Varela, J., Birriel, E., Serna, E., Torres, S., Yaluff, G., de Bilbao, N.V.N.V., Aguirre-López, B., Cabrera, N., Díaz Mazariegos, S., de Gómez-Puyou, M.T.M.T., Gómez-Puyou, A., Pérez-Montfort, R., Minini, L., Merlino, A., Cerecetto, H., González, M., Alvarez, G., 2016. Potent and Selective Inhibitors of Trypanosoma cruzi Triosephosphate Isomerase with Concomitant Inhibition of Cruzipain: Inhibition of Parasite Growth through Multitarget Activity. ChemMedChem 11, 1328-1338. doi:10.1002/cmdc.201500385
